# Methionine enkephalin upregulates toll-like receptors in macrophages to suppress severe fever with thrombocytopenia syndrome virus infection

**DOI:** 10.3389/fimmu.2025.1700988

**Published:** 2025-11-18

**Authors:** Jing Tian, Zhuping Ma, Xiaomeng Wang, Yonggang Li, Yulian Mu, Xiaoli Tao

**Affiliations:** 1 School of Basic Medical Sciences, Jinzhou Medical University, Jinzhou, China; 2 Collaborative Innovation Center for Prevention and Control of Zoonoses, Jinzhou Medical University, Jinzhou, China; 3 School of Basic Medical Sciences, Shenyang Medical College, Shenyang, China; 4 State Key Laboratory of Animal Biotech Breeding, Institute of Animal Science, Chinese Academy of Agricultural Sciences, Beijing, China

**Keywords:** methionine enkephalin, toll-like receptors, macrophages, severe fever with thrombocytopenia syndrome virus, RNA sequencing

## Abstract

**Background:**

Severe fever with thrombocytopenia syndrome virus (SFTSV) is a newly identified tick-borne virus with a high case fatality rate. Currently, no specific antiviral drugs are available for its treatment. Methionine enkephalin (MENK) has the ability to enhance immune cell function nonspecifically. This study investigated the mechanism by which MENK regulates macrophages to exert antiviral effects against SFTSV infection.

**Methods:**

An SFTSV-infected RAW264.7 macrophage model pretreated with MENK was developed. RNA sequencing was employed to analyze transcriptomic alterations and identify differentially expressed genes (DEGs). Subsequent experimental validation based on bioinformatics analysis was performed on key DEGs.

**Results:**

The DEGs between the MENK–SFTSV and SFTSV groups were mainly enriched in several signaling pathways, including the Toll-like receptor (TLR) signaling pathway (ko04620), IL-17 signaling pathway (ko04657), TNF signaling pathway (ko04668), and interaction between viral proteins and cytokines and their receptors (ko04061). Key genes included TLR7, TLR3, TLR8, TLR9, activator protein-1 (AP-1), IL-17RA, TNF-α, IL-1β, CCR5, and CXCL10, all of which successfully docked with the MENK molecule. Compared with the TLR7 agonist imiquimod (IMQ), MENK demonstrated a greater advantage in modulating macrophages to resist SFTSV infection. MENK and the TLR7 agonist imiquimod can upregulate the expressions of TLR7, TLR3, AP-1, and IL-17RA. In addition, MENK significantly upregulated the levels of TLR9, TLR8, IL-1β, TNF-α, CCR5, and CXCL10.

**Conclusion:**

MENK upregulated the expression levels of the pattern recognition receptors and cytokine receptors; promoted the nuclear transcription to modulate cellular immune responses; and increased the levels of the cytokines to induce inflammatory responses against SFTSV infection. Notably, MENK possessed unique advantages and exhibited efficacy comparable to IMQ in restoring antiviral immunity, suggested that MENK emerged as a promising host-directed therapeutic candidate or vaccine prototype against SFTSV infection, warranting further preclinical and clinical investigation.

## 1 Introduction

Severe fever with thrombocytopenia syndrome virus (SFTSV) is a negative-sense single-stranded RNA virus with a genome consisting of three RNA segments, namely, L, M, and S (1). The S segment encodes nonstructural proteins (NSs), which serve as critical virulence factors that suppress host antiviral immune responses through multiple mechanisms, including NF-κB activation 2–mediated pathways, playing a pivotal role in the viral evasion of innate immunity (1). SFTSV causes severe fever with thrombocytopenia syndrome (SFTS), an acute infectious disease that is predominantly endemic to East Asia. In recent years, China and its neighboring regions have experienced sporadic cases and localized outbreaks of SFTS, which poses remarkable public health concerns (2). SFTSV is primarily transmitted through tick bites (3). Following infection, patients typically develop symptoms, including fever, thrombocytopenia, and leukopenia. In severe cases, the disease may progress to multiple organ failure, with case fatality rates ranging from 12% to 30% (4–6). Currently, no specific antiviral drugs are available for the treatment of SFTSV infection. Therefore, in-depth investigations into SFTSV infection mechanisms and host immune defense responses, along with the identification of effective therapeutic targets and intervention strategies, holds considerable clinical importance.

The precise infection mechanisms of SFTSV have not yet been fully elucidated. However, previous studies have demonstrated that SFTSV initially binds to host cell receptor chemokine receptor 2 through its surface glycoprotein Gn; this phenomenon is followed by Gc-mediated membrane fusion between the viral envelope and host cell membrane (7, 8). This process facilitates the release of viral RNA (vRNA) into the cytoplasm of host cells, such as monocytes/macrophages and dendritic cells, initiating viral replication (9, 10). Following SFTSV infection, the excessive activation of the host immune system occurs, leading to immune dysregulation that exacerbates disease progression (11, 12). While the host immune system normally combats viral infection through cytokine and interferon responses, an overactivated immune response may trigger severe inflammatory reactions and organ damage. Clinical investigations have revealed dramatically elevated levels of inflammatory cytokines and chemokines, particularly IL-1β, TNF-α, IL-6, IFN-I, G-CSF, and CXCL10, in the sera of patients with SFTS, with significantly higher concentrations observed in patients with severe SFTS than in those with mild SFTS (13, 14).

Toll-like receptors (TLRs) serve as the body’s primary defense mechanism by recognizing both pathogen-expressed molecules and host-derived molecules released from damaged or dying cells (15). To date, 13 TLRs (TLR1-TLR13) have been identified in mammals and humans. Activation of the TLR pathway leads to the secretion of pro-inflammatory cytokines, such as IL-1, IL-6, and TNF-α, as well as type I interferons. Various TLRs, including TLR2, TLR3, TLR4, TLR6, TLR7, TLR8, and TLR9, may have potential importance in COVID-19 infection. TLRs could serve as potential targets for controlling infection at the early stage of the disease and for developing vaccines against SARS-CoV-2 (16). Xu Z et al. (17) reported that emodin from aloe vera could significantly inhibit porcine reproductive and respiratory syndrome virus (PRRSV) infection in Marc-145 cells and porcine alveolar macrophages *in vitro* by activating TLR3. Felipe VLJ et al. (18) found that TLR2, TLR7, and TLR8 on monocytes, as well as TLR3 and TLR7 in monocyte-derived macrophages, recognize chikungunya virus, leading to the increased production of proinflammatory cytokines and levels of elevated IFN-I, thereby enhancing the antiviral state of cells. Nevertheless, the interaction mechanism between macrophage immune responses and SFTSV replication remains unclear. Only a few studies have reported that the envelope glycoprotein Gn of SFTSV is a potent inhibitor of the cGAS–STING pathway, preventing the nuclear accumulation of interferon regulatory factor 3 and p65, thereby suppressing downstream innate immune signaling. Consequently, enhancing the innate antiviral immune function of macrophages for preventing and treating SFTSV infection has become a key research focus.

Methionine enkephalin (MENK), an endogenous opioid peptide, is widely distributed in neural, endocrine, and immune cells. It not only possesses physiological functions, such as analgesia and mood regulation, but also plays an important role in immune modulation (19). By binding to opioid receptors on immune cell surfaces, MENK regulates the activity and function of T cells, dendritic cells, macrophages, and other immune cells, thereby enhancing host immune defense capabilities (20). Our previous work revealed that MENK potentiates macrophage antiviral responses against the influenza virus by upregulating the TLR4–NF-κB p65 signaling pathway, enhancing opsonic receptors (FcγR/CR3) and CCL4 chemokine signaling, which promotes phagocytosis, antigen presentation, and viral clearance (21, 22). These findings prompted us to investigate whether MENK could confer protection against SFTSV infection through immunomodulation. In this study, we established an SFTSV-infected RAW264.7 macrophage model with MENK prophylactic treatment. By using RNA sequencing (RNA-seq) transcriptomic profiling combined with the experimental validation of key targets, we systematically examined the immunoregulatory mechanisms of MENK. Comparative studies with TLR7 agonists elucidated the critical role of TLR in MENK-mediated anti-SFTSV responses. Our study aims to provide a foundation for the potential use of MENK as a novel SFTSV therapeutic or vaccine adjuvant and to offer new insights for developing innovative clinical strategies against SFTSV infection.

## 2 Materials and methods

### 2.1 Study subjects

The murine macrophage cell line RAW264.7 was purchased from the Cell Resource Center of the Chinese Academy of Sciences (Shanghai, China). SFTSV (virus ID: SDYY007) was provided by the Institute of Microbiology, Chinese Academy of Sciences.

### 2.2 Antibodies and reagents

MENK (purity ≥ 99%) was obtained from American Peptide Company Inc. (the USA). Imiquimod (IMQ, Cat# T0134) was purchased from TargetMol (the USA). PrimeScript™ RT Reagent Kit with gDNA Eraser (Cat# RR047A) and TB Green^®^ Premix Ex Taq™ II (RR820A) were acquired from TaKaRa (Japan). Antibodies against TLR7 (Cat# DF6173), TLR8 (Cat# DF6426), TLR9 (Cat# DF2970), IL-1β (Cat# AF5103), TNF-α (Cat# AF7014), IL-10 (Cat# DF6894), β-actin (Cat# T0022), and Alexa Fluor 488–conjugated antirabbit secondary antibody (Cat# S0018) were sourced from Affinity Biosciences (the USA). HRP-conjugated antimouse (Cat# A0350) and antirabbit (Cat# A0352) secondary antibodies, NP-40 lysis buffer (Cat# P0013F), and BCA protein assay kit (Cat# P0012) were procured from Beyotime (China). The anti-SFTSV NS antibody was prepared by the Pathogen Biology Laboratory at Jinzhou Medical University (23).

### 2.3 Experimental group design

**Figure 1:**
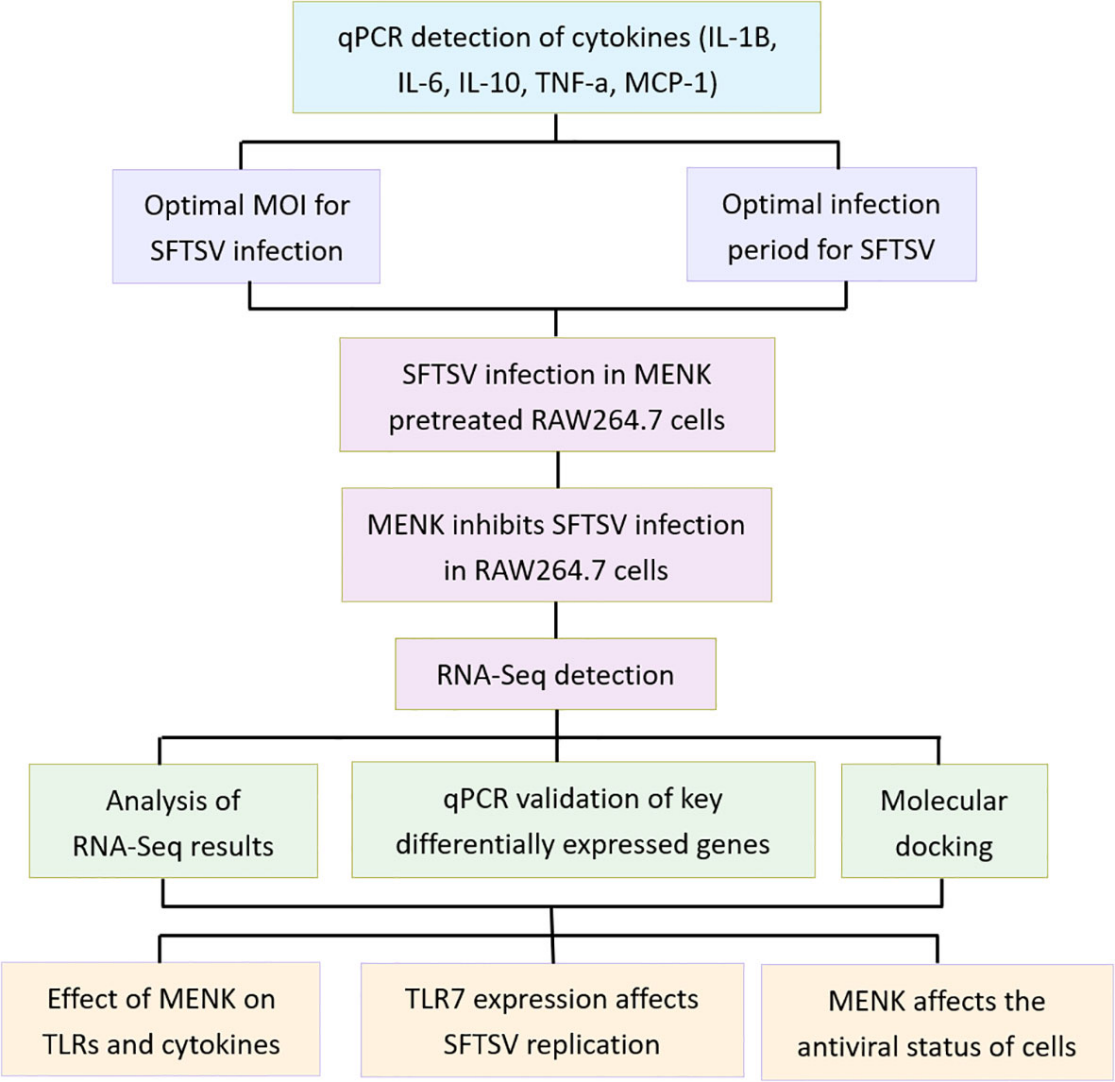
A flowchart of the experimental design.

This study comprised three primary experimental groups: the Control, SFTSV, and MENK–SFTSV groups. The Control group consisted of RAW264.7 cells cultured in DMEM maintenance medium for 48 h. Cells in the SFTSV group were infected with SFTSV at a multiplicity of infection (MOI) of 200 and incubated for 1 h at 37°C with 5% CO_2_. Incubation was followed by removing the viral supernatant, washing cells with PBS, and culturing cells in DMEM maintenance medium for 48 h. RAW264.7 cells in the MENK–SFTSV group were pretreated with MENK (10 mg/ml, 2 ml/well) for 48 h prior to viral challenge. After being washed with PBS, cells were infected with SFTSV at an MOI of 200 for 1 h at 37°C with 5% CO_2_, then subjected to viral supernatant removal, PBS washing, and final culture in DMEM maintenance medium for 48 h before sample collection. An additional IMQ–SFTSV group was included to investigate further whether MENK enhanced macrophage antiviral function via TLR7. In this group, RAW264.7 cells were pretreated with IMQ (8 µg/ml, 2 ml/well) 24 h before virus challenge and incubated at 37°C with 5% CO_2_. Prior to infection, cells were washed with PBS, then infected with SFTSV at an MOI of 200 and incubated for 1 h. After the virus inoculum was removed and cells were washed with PBS, cells were added with DMEM maintenance medium and cultured for another 48 h before sample collection. The follow-up experimental design was divided into dry lab section and wet lab section. A flowchart of the experimental design is presented in **Figure 1**.

### 2.4 Dry lab section

#### 2.4.1 RNA-seq and data analysis

Total RNA was extracted by using Trizol reagent, and RNA integrity was verified through 1.0% agarose gel electrophoresis. RNA quality and quantity were assessed by employing NanoPhotometer^®^ spectrophotometry and Qubit^®^ 2.0 fluorometry. High-quality RNA samples were sent to Sangon Biotech for library preparation with the VAHTSTM mRNA-seq V2 Library Prep Kit (Illumina^®^). The sequencing was performed on the MGI Tech DNBSEQ-T7 platform. The data size is 6Gb (~20M reads per sample). The sequencing depth ranged from 6.04x to 8.45x. Raw sequencing data underwent quality assessment through FastQC, then filtered to obtain clean data. A random subset of 10,000 reads from the clean data was aligned against the NCBI NT database, with alignment statistics generated by using RSeQC. Differentially expressed genes (DEGs) were identified by imposing thresholds of qValue ≤ 0.05 and |log2FoldChange| ≥ 1, requiring a minimum TPM value of ≥5 in at least one comparison group. DEGs were visualized by using volcano plots to display expression patterns and regulation directions between sample groups. Gene Ontology (GO) annotation was employed to examine the functional distributions of DEGs, revealing sample differences at the functional level. Functional enrichment analyses were performed by using the GO and Kyoto Encyclopedia of Genes and Genomes (KEGG) (24). GO annotation results were presented in the form of bar plots, whereas the findings of GO enrichment and KEGG pathway analyses were visualized by using bubble plots.

#### 2.4.2 Molecular docking

On the basis of the relevant pathways identified through GO and KEGG enrichment analyses, key proteins were selected for molecular docking studies. The interactions between MENK and critical small-molecule compounds were predicted by utilizing Autodock Vina software. Autodock Vina determines the optimal binding conformation and corresponding binding energy (25). The docking results between chemical components and key targets were subsequently visualized with PyMOL software, providing a clear visualization of the interaction details at the molecular level. The protein structures of MENK and the core proteins were obtained from the Protein Data Bank (PDB). The 3D conformations of the complexes formed by their binding were predicted using the ClusPro website, and the minimum binding energies for the relevant structures were obtained (26). Finally, their protein-binding sites were demonstrated using PyMol.

### 2.5 Wet lab section

#### 2.5.1 Western blot analysis

Total protein was extracted from RAW264.7 cells by using NP40 lysis buffer containing PMSF, and protein concentrations were determined by employing a BCA assay kit. Proteins were separated through SDS–PAGE and transferred onto PVDF membranes. The membranes were blocked with 5% skimmed milk at room temperature for 2 h. Subsequently, the membranes were incubated overnight at 4 °C with primary antibodies diluted in antibody dilution buffer. These antibodies included anti-TLR7 (1:1000), anti-TLR8 (1:1000), anti-TLR9 (1:1000), anti-IL-1β (1:1000), anti-TNF-α (1:1000), anti-IL-10 (1:1000), anti-NS (1:3000), and anti-β-actin (1:5000). Following primary antibody incubation, the membranes were washed and incubated with appropriate secondary antibodies. After additional washing steps, target protein bands were visualized through chemiluminescence detection, and images were acquired for densitometric analysis.

#### 2.5.2 Immunofluorescence assay

RAW264.7 cells were subjected to supernatant removal, washed three times with PBS, and fixed with 4% paraformaldehyde for 30 min. Following three additional PBS washes, 500 μl of 0.1% Triton X-100 was added to each well for 10–15 min of permeabilization, after which cells were washed three times with PBS. Subsequently, 500 μl of 1% BSA was added to each well, and cells were incubated at room temperature for 1 h for blocking. After the BSA solution was removed, cells were added with 200 μl of diluted NS antibody (1:800) and incubated at 37 °C for 1 h. Cells were then washed five times with PBS, then incubated with 200 μl of Alexa Fluor 488–conjugated antirabbit secondary antibody at 37 °C for 1 h. After five final PBS washes, cells were mounted with antifade mounting medium containing DAPI and visualized under a fluorescence microscope. The expression and localization of target antigens were determined on the basis of the location and intensity of fluorescent signals.

#### 2.5.3 Quantitative real-time PCR

Total RNA was extracted from cells by using Trizol reagent. This step was followed by the measurement of RNA concentration and purity. cDNA was then synthesized by employing PrimeScript™ RT Reagent Kit. Primer sequences were designed by utilizing the online NCBI Blast tool (https://blast.ncbi.nlm.nih.gov/) (**Supplementary Table S1**). qPCR was performed with TB Green^®^ Premix Ex Taq™ II (Tli RNaseH Plus) on Applied Biosystems 7500 Fast Real-Time PCR System (ABI, the USA). Relative mRNA expression levels were calculated through the 2^−ΔΔCT^ method (27).

#### 2.5.4 CCK8 assay

RAW264.7 cells were seeded in 96-well plates at a density of 5,000 cells per well. On Day 2, cells were treated with the TLR7 agonist IMQ at varying concentrations (0, 0.5, 1, 2, 4, 8, and 16 μg/ml) for either 24 or 48 h. After being washed with PBS, each well received 100 μl of serum-free DMEM supplemented with 10 μl of CCK-8 reagent. After thorough mixing, plates were incubated at 37°C for 1 h. Cell viability was then assessed by measuring absorbance at 450 nm with a microplate reader.

#### 2.5.5 Statistical analysis

All data were expressed as mean ± SD and evaluated through two-sample independent t-tests and one-way analysis of variance. GraphPad Prism 10 software was used for statistical analysis and graphing. Each experiment in this study was conducted in triplicate. Differences were considered statistically significant at *P < 0.05, **P < 0.01, and ***P < 0.001.

## 3 Results

### 3.1 Optimal conditions for the SFTSV infection of RAW264.7 cells

**Figure 2:**
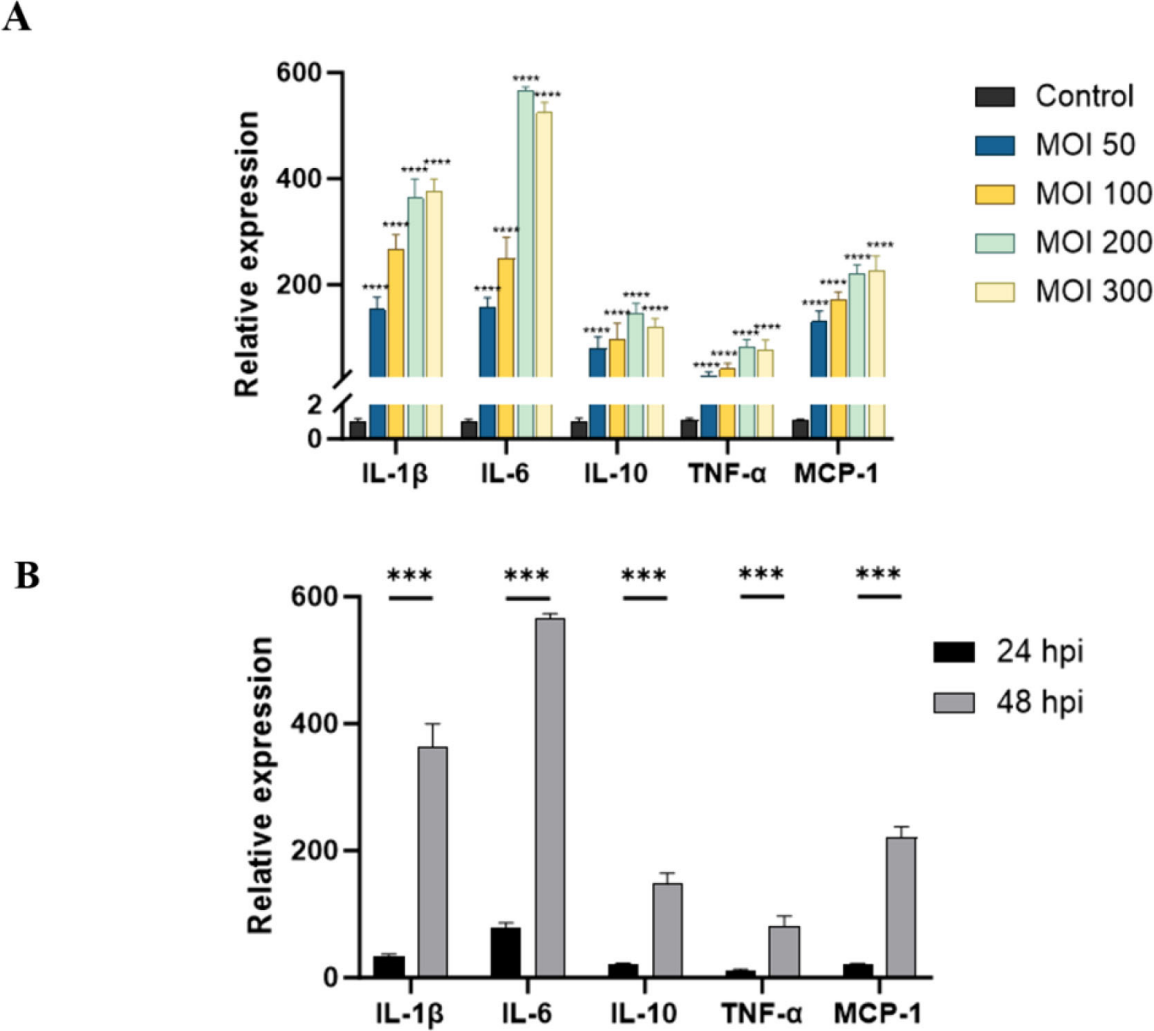
Effects of SFTSV infection at different MOI values and times on macrophage immune functions. **(A)** Changes in cytokines detected by qPCR in RAW264.7 cells infected with SFTSV at different MOI values for 48 (h). Compared with the Control group, ****P < 0.0001 **(B)** qPCR analysis of cytokine changes in macrophages infected with SFTSV (MOI = 200) for 24 h or 48 h. 24 hpi group vs. 48 hpi group: ***P < 0.001.

We infected RAW264.7 cells with SFTSV at the MOI values of 50, 100, 200, and 300 for 48 h. qPCR results demonstrated that the mRNA levels of IL-1β, IL-6, IL-10, TNF-α, and MCP-1 in SFTSV-infected RAW264.7 cells were significantly elevated compared with those in the control cells (P < 0.001). The expression levels of IL-6, IL-10, and TNF-α in RAW264.7 cells peaked at MOI 200, while IL-1β and MCP-1 reached their highest levels at MOI 300 (**Figure 2A**). Cell viability, as determined by CCK-8 assay, was not significantly affected by SFTSV infection at 48 hours, with the exception of the MOI 300 group (P < 0.001) (**Supplementary Figure S1**). Therefore, the MOI of 200 was selected for the infection of RAW264.7 cells in subsequent experiments.

Following the infection of RAW264.7 cells with SFTSV at the MOI of 200 for 24 or 48 h, the levels of IL-1β, IL-6, IL-10, TNF-α, and MCP-1 were measured by using qPCR. Notably, all examined cytokines showed significantly higher levels at 48 h postinfection than at 24 h postinfection (P < 0.001) (**Figure 2B**). On the basis of these findings, SFTSV infection was conducted with the MOI of 200 for 48 h in subsequent experiments.

### 3.2 Morphological changes in RAW264.7 cells

**Figure 3:**
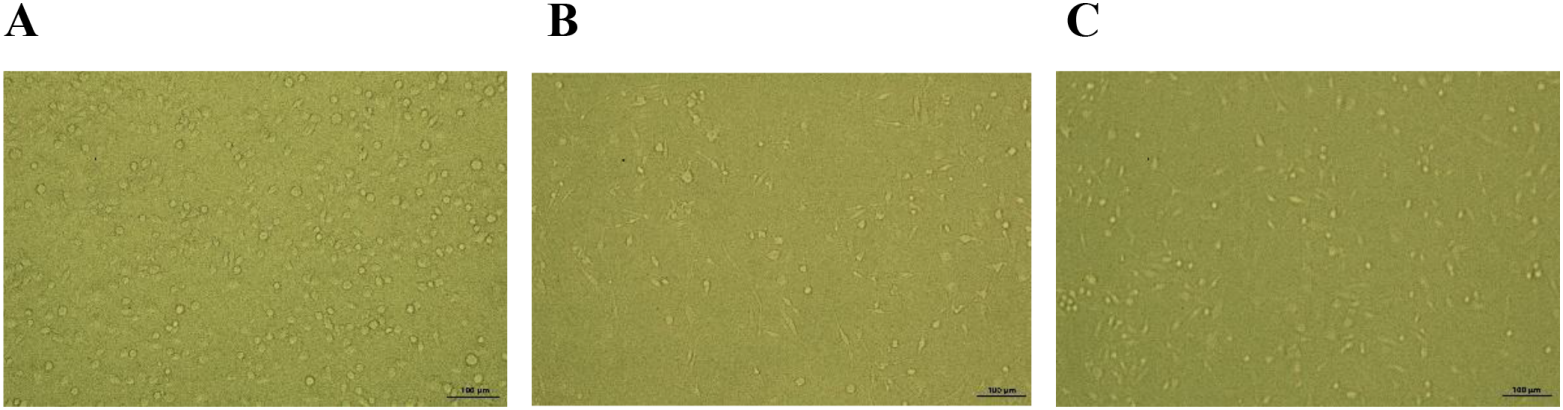
Morphology of RAW264.7 cells under light microscopy (200×). **(A)** Control group, **(B)** SFTSV group, **(C)** MENK–SFTSV group.

Under optical microscopy, RAW264.7 cells in the Control group predominantly exhibited a round or oval shape, with small and translucent morphology. By contrast, cells in the SFTSV group displayed enlarged cell volumes and irregular shapes. These changes were accompanied with the appearance of vacuoles and granular substances in the cytoplasm. Meanwhile, cells in the MENK–SFTSV group mostly appeared elongated and spindle-shaped and exhibited pseudopodia formation and occasional intracellular vacuoles and granular particles (**Figure 3**).

### 3.3 MENK inhibited SFTSV replication in RAW264.7 cells

**Figure 4:**
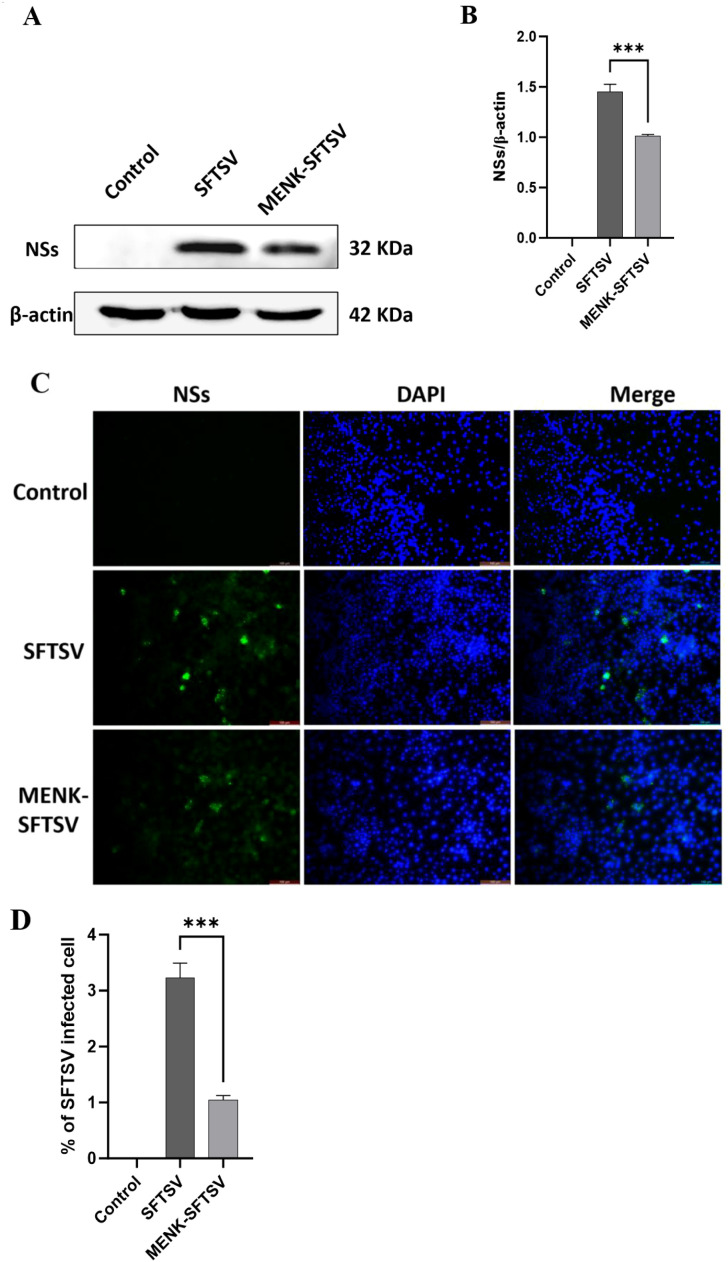
MENK inhibited SFTSV replication in RAW264.7 cells. **(A)** Western blot analysis of NS expression in the Control, SFTSV, and MENK–SFTSV groups. **(B)** Grayscale analysis histogram of SFTSV NSs. **(C)** Immunofluorescence detection of NS expression (400×). Green fluorescence indicates SFTSV NSs, and blue fluorescence represents DAPI-stained nuclei. **(D)** The Percentage of infected RAW264.7 cells in SFTSV, and MENK–SFTSV groups was measured by immunofluorescence images. The image-associated fluorescence was measured using Image J software. Each sample was observed under 400× magnification for 10 fields of view. Percentage of infected cells = (Number of infected cells)/(Total number of cells). SFTSV group vs. MENK–SFTSV group, ***P < 0.001.

In accordance with our established protocol (21), RAW264.7 cells in the MENK–SFTSV group were pretreated with 10 mg/ml MENK for 48 h prior to infection with SFTSV at the MOI of 200 for an additional 48 h. Western blot analysis revealed that NS expression in the MENK–SFTSV group had significantly reduced compared with that in the SFTSV group (P < 0.001) (**Figures 4A, B**). Immunofluorescence assays confirmed that MENK pretreatment substantially decreased the number of SFTSV-infected cells (P < 0.001) (**Figures 4C, D**). These results indicated that MENK pretreatment enhanced macrophage antiviral activity, thereby effectively suppressing SFTSV replication within macrophages.

### 3.4 Quality control assessment of RNA-seq data

RNA-seq raw data were subjected to quality control analysis by using Trimmomatic software. As shown in **Supplementary Table S2**, the processed clean data ranged from 36,372,334 reads per sample to 53,022,330 reads per sample. The sequencing quality metrics demonstrated excellent results, with Q20 score ≥ 98.32% and Q30 score ≥ 95.43%. No significant contamination from other species was detected, and ≥91.31% of the sequencing reads were successfully mapped to the reference genome. These results confirmed the high reliability of our sequencing data for subsequent analyses.

### 3.5 Results of differential gene expression analysis

**Figure 5:**
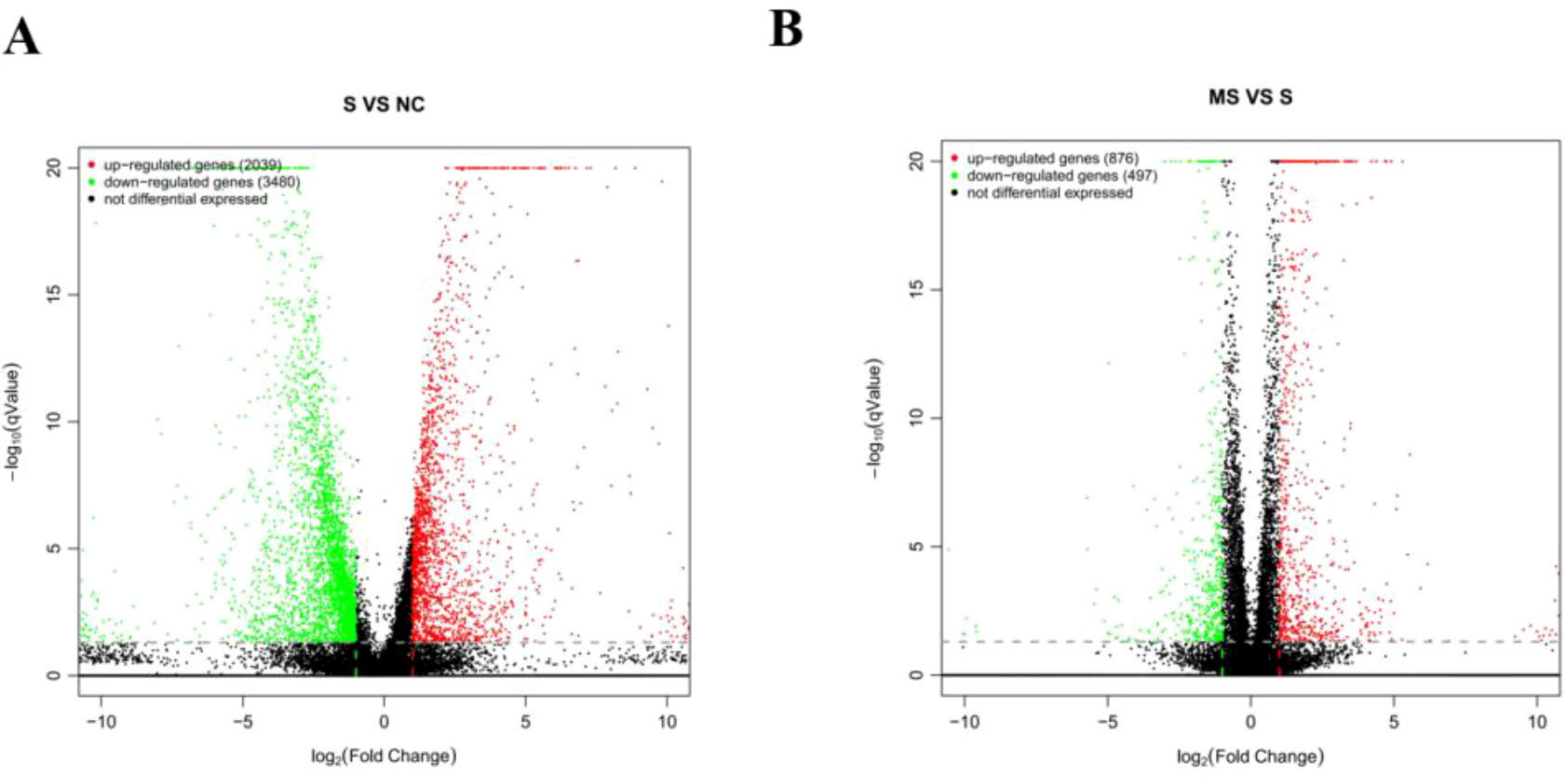
Volcano plot of DEGs. **(A)** Volcano plot of DEGs between the Control and SFTSV groups. **(B)** Volcano plot of DEGs between the SFTSV and MENK–SFTSV groups.

Volcano plots were used for the visualization and analysis of DEGs (**Figure 5**). Under the criteria of qValue < 0.05 and |log2FoldChange| > 15,519 DEGs were identified between the SFTSV and Control groups, with 2,039 genes being upregulated and 3,480 genes being downregulated. Comparison between the MENK–SFTSV and SFTSV groups revealed that 1373 DEGs met the above criteria. These DEGs included 876 upregulated genes and 497 downregulated genes.

### 3.6 GO annotation analysis

**Figure 6:**
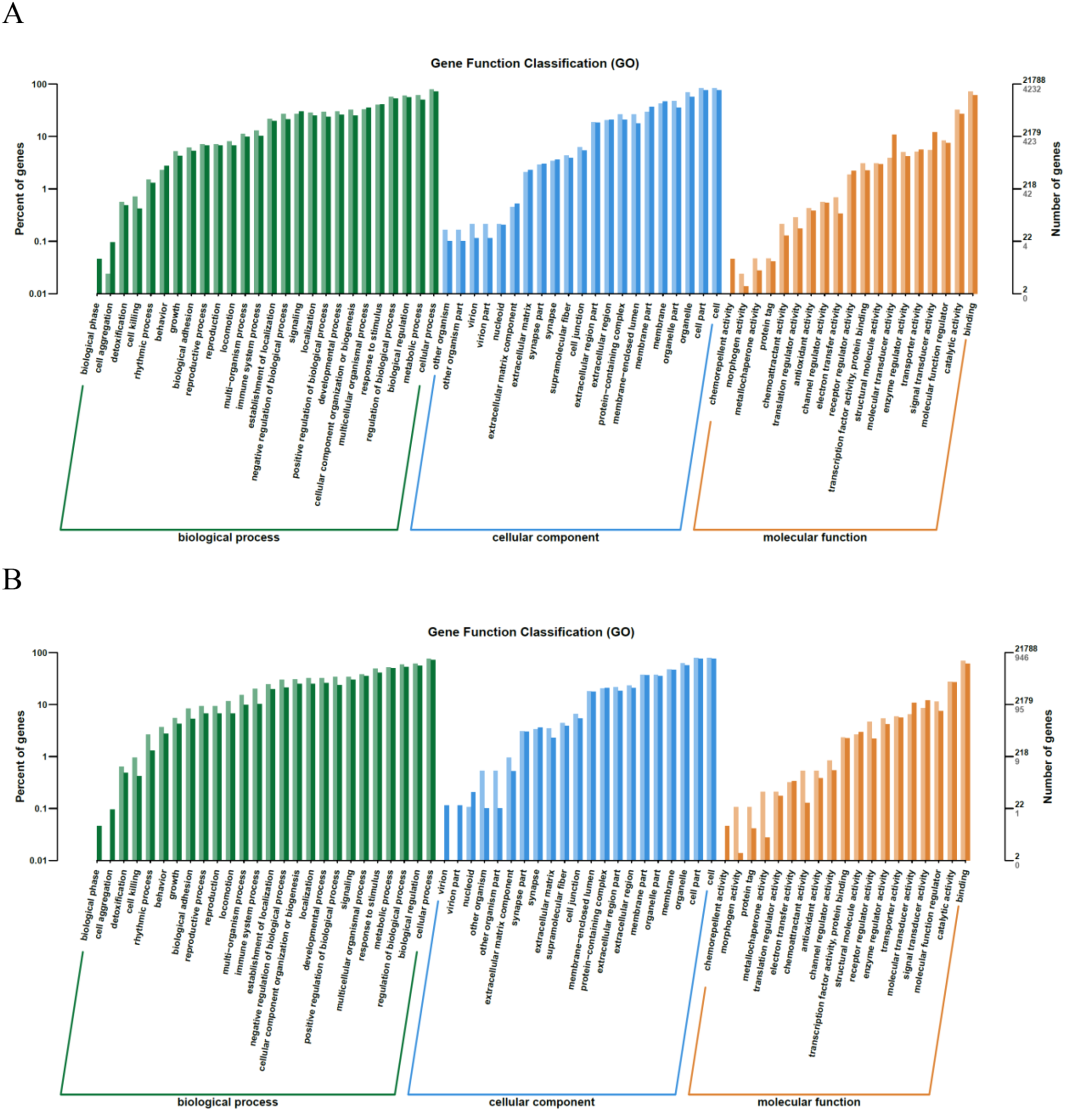
Bar plot of GO annotation categories. **(A)** GO annotation categories for the SFTSV and Control groups. **(B)** GO annotation categories for the MENK–SFTSV and SFTSV groups. The left vertical axis (percentage of genes) represents the ratio of DEGs to all genes. Light-colored bars represent DEGs, whereas dark-colored bars represent all genes. On the vertical axis, gray numbers indicate DEGs, and black numbers indicate all genes.

We statistically analyzed the distribution of DEGs in annotated functions for their functional description and classification (**Figure 6**). In the GO analysis between the SFTSV and Control groups, DEGs in the BP category were involved in cell killing, proliferation, and migration. The CC category included virion part, protein-containing complex, membrane-enclosed lumen, and organelle. The MF category encompassed enhanced chemokine activity and electron transfer activity (**Figure 6A**). In the comparison between the MENK–SFTSV and SFTSV groups, the DEGs in the BP category were found to be associated with cell killing, biological adhesion, immune system processes, and signal transduction. The CC category encompassed extracellular matrix components, cell junctions, and supramolecular fibers. The MF category was related to protein tagging, antioxidant activity, and receptor regulator activity (**Figure 6B**).

### 3.7 GO enrichment analysis

**Figure 7:**
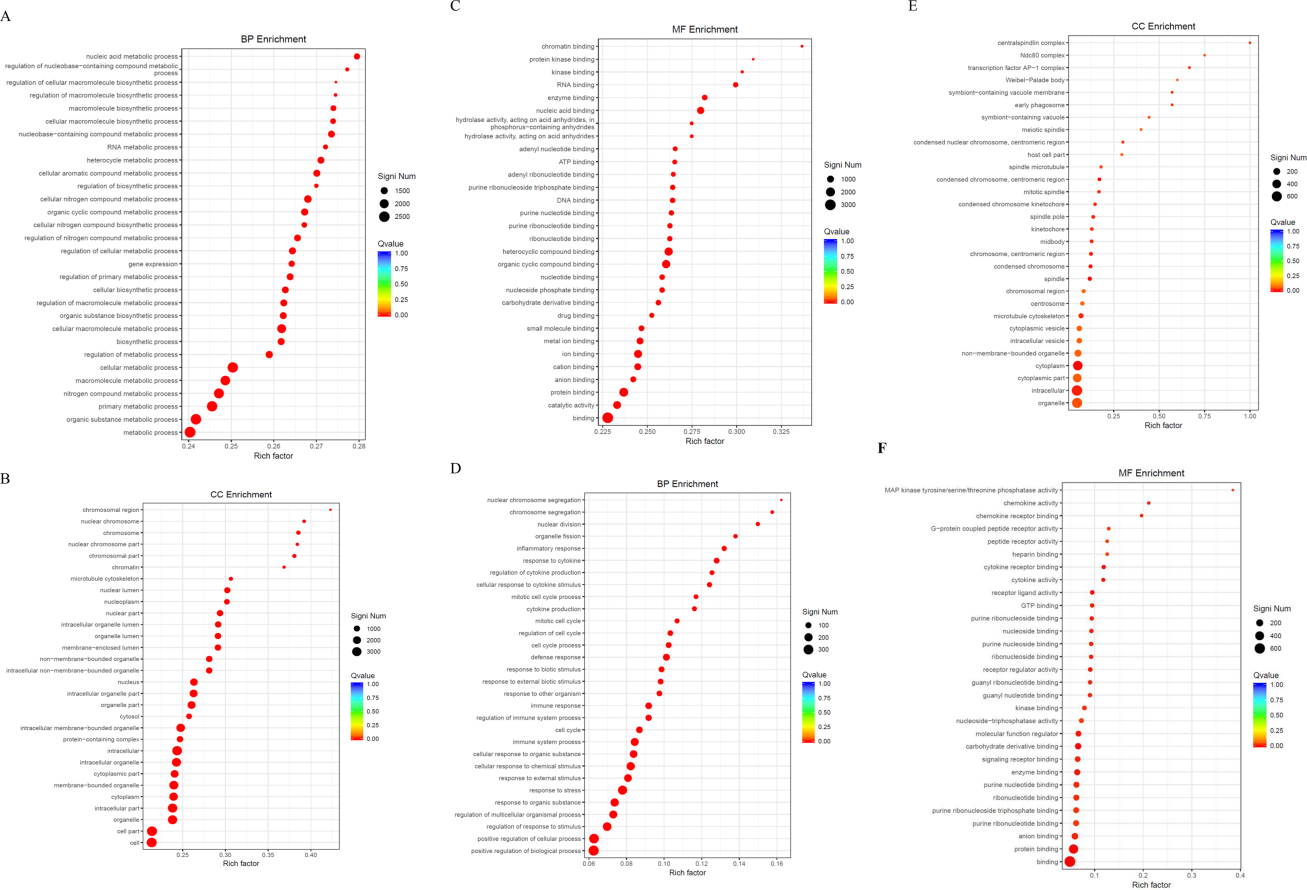
GO enrichment analysis. **(A)** Bubble plot of DEGs in the BP category for the SFTSV and Control groups. **(B)** Bubble plot of DEGs in the CC category for the SFTSV and Control groups. **(C)** Bubble plot of DEGs in the MF category for the SFTSV and Control groups. **(D)** Bubble plot of DEGs in the BP category for the MENK–SFTSV and SFTSV groups. **(E)** Bubble plot of DEGs in the CC category for the MENK–SFTSV and SFTSV groups. **(F)** Bubble plot of DEGs in the MF category for the MENK–SFTSV and SFTSV groups. The color gradient of the dots represents the magnitude of the Q-value, with intense red coloration indicating small Q-values. The size of each dot corresponds to the number of DEGs within each functional term, where large dots denote terms containing large numbers of DEGs.

We conducted GO functional enrichment analysis to determine the main biological functions of DEGs. The GO enrichment analysis between the SFTSV and Control groups (**Table 1**) revealed that in the BP category, DEGs were significantly enriched in biological regulation and macromolecule metabolic processes (**Figure 7A**). In the CC classification, DEGs were primarily enriched in cytoplasmic regions and organelles (**Figure 7B**). In the MF category, DEGs showed significant enrichment in protein and ion binding activities (**Figure 7C**).

**Table 1:** Significant GO enrichment terms in SFTSV and control groups.

GO ID	Term	Ontology	Significant	qValue
GO:0065007	biological regulation	BP	2550/4084	3.29E-09
GO:0043170	macromolecule metabolic process	BP	2180/4084	3.18E-28
GO:0043226	organelle	CC	2962/4135	7.92E-29
GO:0005737	cytoplasm	CC	2402/4135	7.92E-29
GO:0005515	protein binding	MF	1887/4059	1.05E-27
GO:0043167	ion binding	MF	1443/4059	8.00E-25

The GO enrichment analysis of the MENK–SFTSV and SFTSV groups (**Table 2**) revealed that the DEGs in the BP category were enriched in biological regulation, response to stimulus, and cellular macromolecule metabolic process (**Figure 7D**). The DEGs in the CC category were primarily enriched in the cytoplasm and organelles (**Figure 7E**). The DEGs in the MF category were notably enriched in enzyme activity, ion binding, and protein binding (**Figure 7F**).

**Table 2:** Significant GO enrichment terms in SFTSV-MENK and SFTSV groups.

GO ID	Term	Ontology	Significant	qValue
GO:0065007	biological regulation	BP	372/909	1.48E-02
GO:0050896	response to stimulus	BP	388/909	2.47E-05
GO:0044260	cellular macromolecule metabolic process	BP	586/909	1.48E-02
GO:0005737	cytoplasm	CC	515/929	2.30E-04
GO:0043226	organelle	CC	595/929	3.37E-02
GO:0019899	enzyme binding	MF	127/907	3.37E-02
GO:0043168	anion binding	MF	159/907	7.44E-03
GO:0005515	protein binding	MF	451/907	9.16E-09

### 3.8 KEGG enrichment analysis

**Figure 8:**
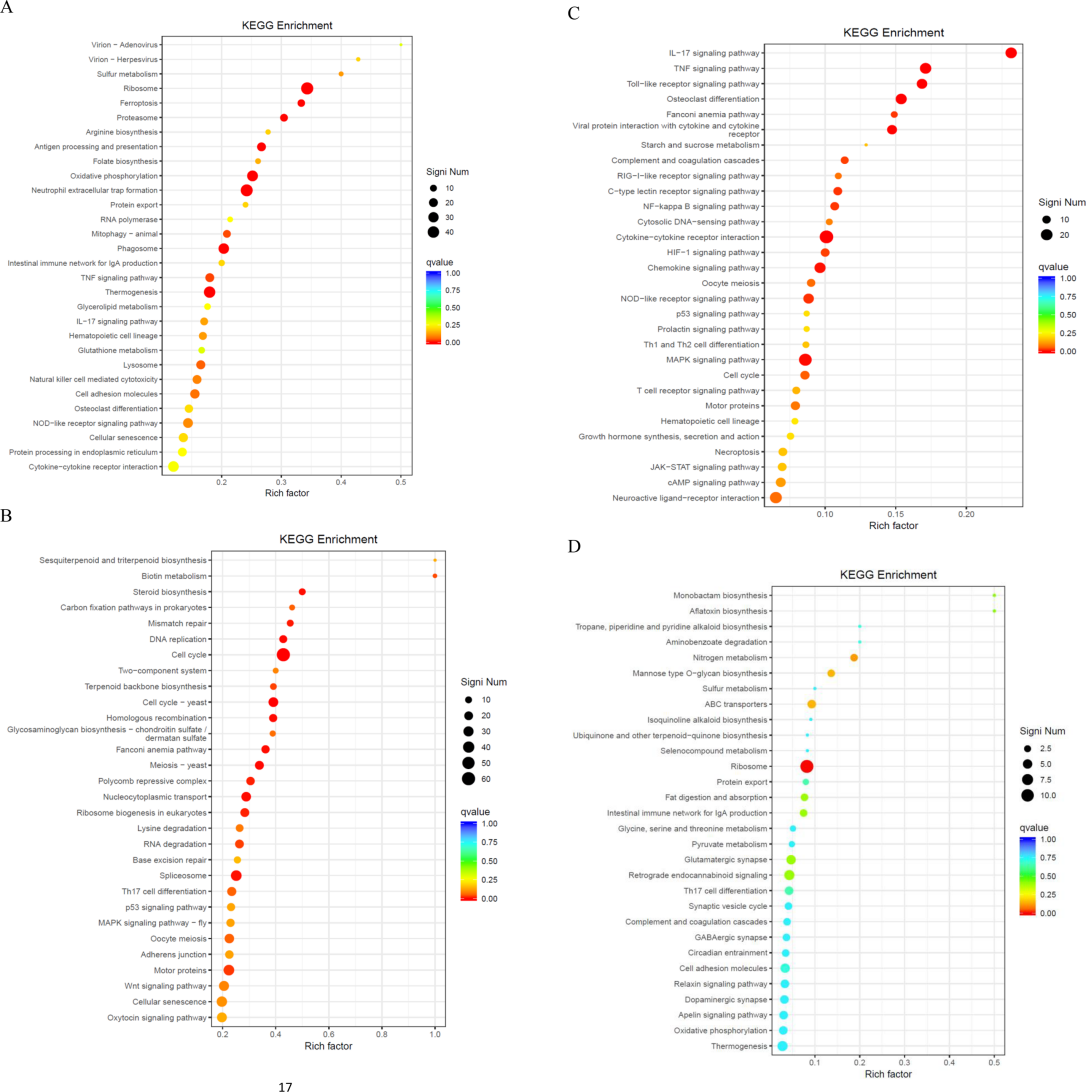
KEGG enrichment analysis. **(A)** Bubble plot of the enrichment analysis of upregulated DEGs in the SFTSV and Control groups. **(B)** Bubble plot of the enrichment analysis of downregulated DEGs in the SFTSV and Control groups. **(C)** Bubble plot of the enrichment analysis of upregulated DEGs in the MENK–SFTSV and MENK groups. **(D)** Bubble plot of the enrichment analysis of downregulated DEGs in the MENK–SFTSV and MENK groups.

We performed KEGG enrichment analysis to annotate DEGs in metabolic pathways, thus providing insights into the effect of gene expression changes on biological processes. This analysis elucidated the functional roles of DEGs in various metabolic pathways. The KEGG pathway enrichment analysis of the SFTSV and Control groups revealed significant involvement in ferroptosis (ko04216), antigen processing and presentation (ko04612), oxidative phosphorylation (ko00190), neutrophil extracellular trap formation (ko04613), and cell cycle (ko04110) (**Figures 8A, B**; **Table 3**). In the ko04613 pathway, FcγRI and FcγRIV were upregulated, suggesting that macrophages recognize viral particles through surface Fc receptors for subsequent clearance following SFTSV infection. Conversely, TLR2, TLR4, TLR7, and TLR8 were downregulated, indicating that SFTSV may suppress TLR signaling to evade immune responses (**Supplementary Figure S2**).

**Table 3:** Statistics of key KEGG pathways in SFTSV and control groups.

ID	Pathway	qValue
ko4216	Ferroptosis	1.82E-02
ko04612	Antigen processing and presentation	5.71E-03
ko00190	Oxidative phosphorylation	2.36E-03
ko04613	Neutrophil extracellular trap formation	9.60E-04
ko04110	Cell cycle	2.68E-10

The KEGG pathway enrichment analysis of the MENK–SFTSV and SFTSV groups revealed the significant involvement of key signaling pathways, including the IL-17 signaling pathway (ko04657), TNF signaling pathway (ko04668), TLR signaling pathway (ko04620), and viral protein interaction with cytokines and cytokine receptors (ko04061) (**Figures 8C, D**; **Table 4**). Elevated levels of IL-17RA in ko04657 (**Supplementary Figure S3**), along with the increased expression levels of TLR3, TLR7, TLR8, and TLR9 in ko04620 (**Supplementary Figure S4**), suggested that MENK enhanced the recognition of pathogen-associated molecular patterns by macrophages, thereby promoting innate immune activation and accelerating viral clearance. Furthermore, the upregulation of TNF and CXCL10 in ko04668 (**Supplementary Figure S5**), as well as the increased levels of IL-1, TNF-α, and CCR5 in ko04061 (**Supplementary Figure S6**), indicated that MENK strengthened macrophage antiviral responses by enhancing the chemotactic recruitment of immune cells and promoting the secretion of proinflammatory cytokines.

**Table 4:** Statistics of key KEGG pathways in SFTSV-MENK and SFTSV groups.

ID	Pathway	qValue
ko04657	IL-17 signaling pathway	1.06E-07
ko04668	TNF signaling pathway	7.58E-05
ko04620	Toll-like receptor signaling pathway	2.90E-04
ko04380	Osteoclast differentiation	2.90E-04
ko04061	Viral protein interaction with cytokine and cytokine receptor	3.09E-03

### 3.9 qPCR Validation

**Figure 9:**
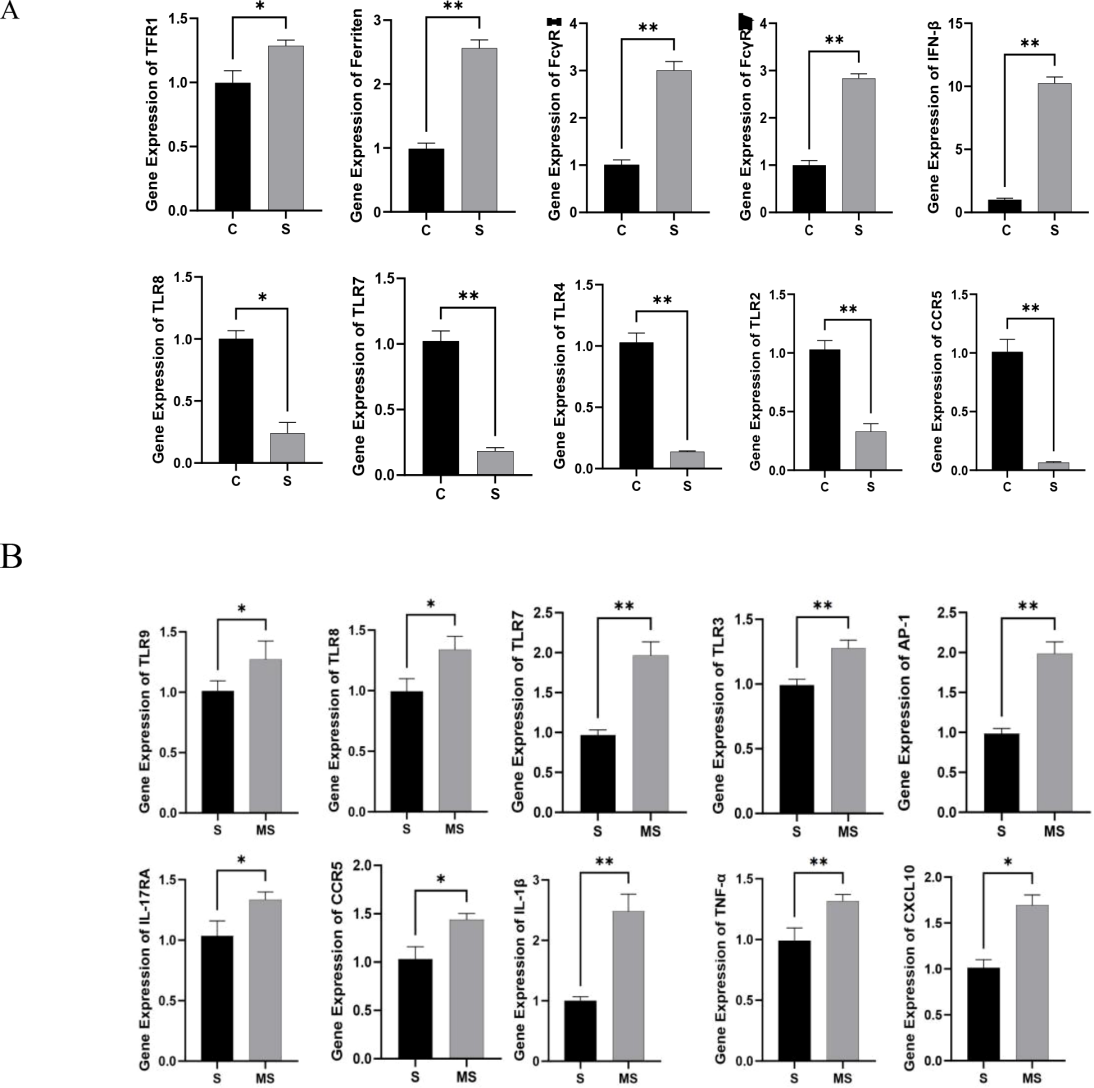
qPCR validation. **(A)** Validation of 10 key DEGs between the SFTSV and Control Groups by qPCR. Compared with the Control group, *P < 0.05, **P < 0.01. **(B)** Validation of 10 key DEGs between the MENK–SFTSV and SFTSV groups by qPCR. Compared with the SFTSV group, *P < 0.05, **P < 0.01.

We selected the key genes TFR1, ferritin, FcγRI, FcγRIV, IFN-β, TLR8, TLR7, TLR4, TLR2, and CCR5 from the KEGG-enriched pathways that showed significant differences between the SFTSV and Control groups to validate the reliability of RNA-seq results. qPCR analysis revealed that compared with the Control group, the SFTSV group exhibited upregulated TFR1, Ferritin, FcγRI, FcγRIV, and IFN-β levels but downregulated TLR8, TLR7, TLR4, TLR2, and CCR5 levels (**Figure 9A**). These findings were consistent with the trends of RNA-seq data (**Supplementary Table S3**).

Comparative analysis between the MENK–SFTSV and SFTSV groups revealed that RAW264.7 cells in the MENK–SFTSV group exhibited upregulated expression levels of TLR9, TLR8, TLR7, TLR3, IL-17RA, activator protein-1 (AP-1), TNF-α, IL-1β, CCR5, and CXCL10 (**Figure 9B**). These qPCR results were consistent with the transcriptomic trends observed in the RNA-seq data (**Supplementary Table S4**).

### 3.10 Molecular docking analysis

**Figure 10:**
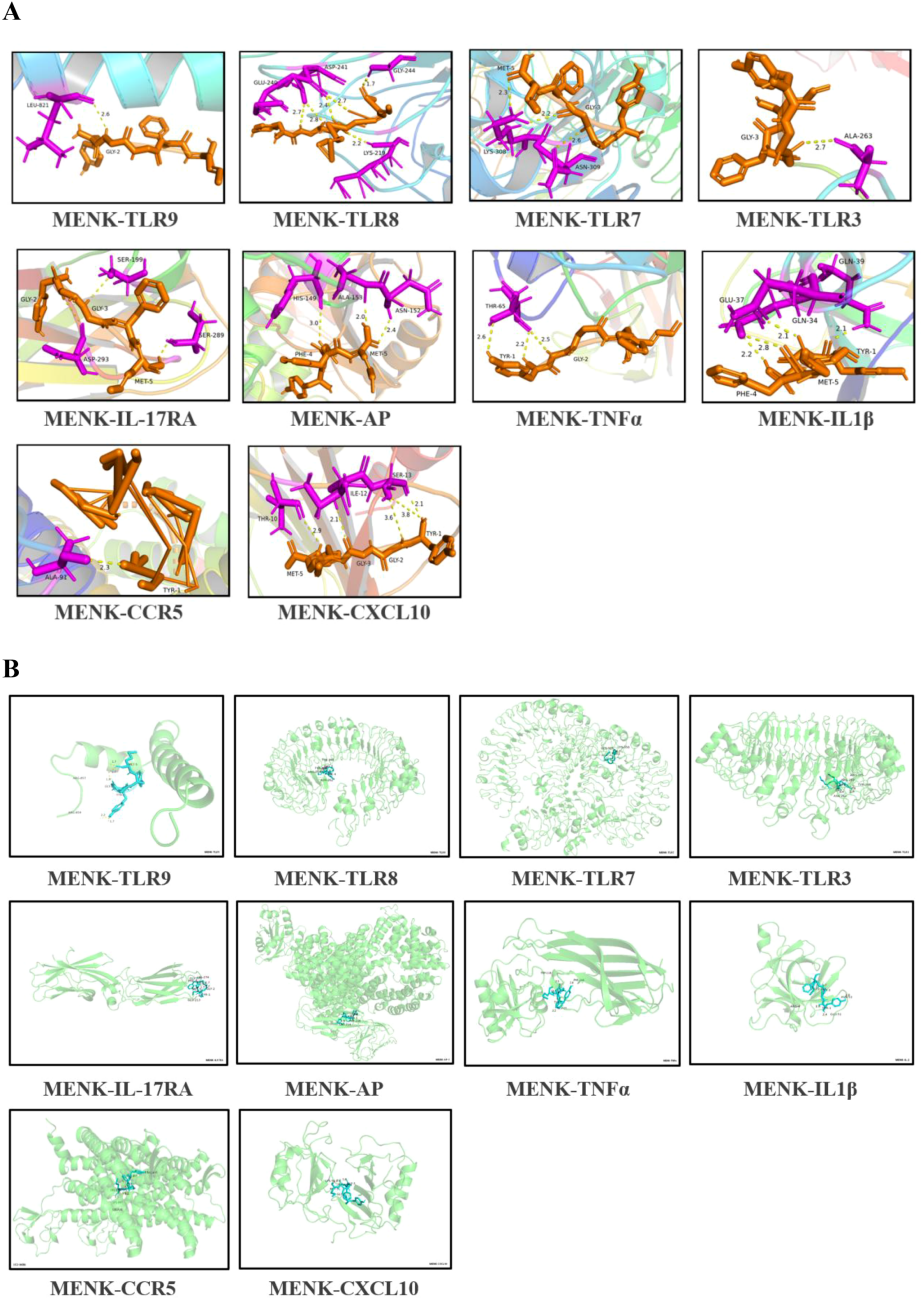
Molecular docking simulation of MENK with core proteins. **(A)** MENK is represented by orange stick models, and the protein molecules at the docking site are presented as blue stick models. Connected hydrogen bonds are indicated by yellow dotted lines. **(B)** A 3D model was generated to simulate the binding states of MENK (blue) and core proteins (green). The model was obtained from the PDB database using the ClusPro website, which predicts protein-protein interactions and complex structures.

On the basis of the KEGG enrichment pathways, we evaluated the interactions between MENK and 10 core proteins, namely, TLR9, TLR8, TLR7, TLR3, IL-17RA, AP-1, TNF-α, IL-1β, CCR5, and CXCL10. Computational analysis revealed favorable binding affinities between MENK and the core proteins. The binding energies of MENK with these core proteins were as follows: TLR9: −5.93 kcal/mol, TLR8: −9.16 kcal/mol, TLR7: −3.37 kcal/mol, TLR3: −4.64 kcal/mol, IL-17RA: −7.78 kcal/mol, AP-1: −6.49 kcal/mol, TNF-α: −5.42 kcal/mol, IL-1β: −6.65 kcal/mol, CCR5: −4.34 kcal/mol, and CXCL10: −7.26 kcal/mol. Successful molecular docking with MENK was confirmed for all target proteins (**Figure 10A**). The protein structures of MENK and 10 core proteins were obtained from the PDB, and the 3D conformations of the complexes formed by their binding were predicted using the Cluspro website and the minimum binding energies were obtained for the relevant structures (**Supplementary Table S5**), and finally their protein-binding sites were demonstrated by Pymol (**Figure 10B**). All of these results support that the protein of MENK and 10 core proteins bind to each other.

### 3.11 Effects of MENK on TLRs and cytokines in RAW264.7 cells

**Figure 11:**
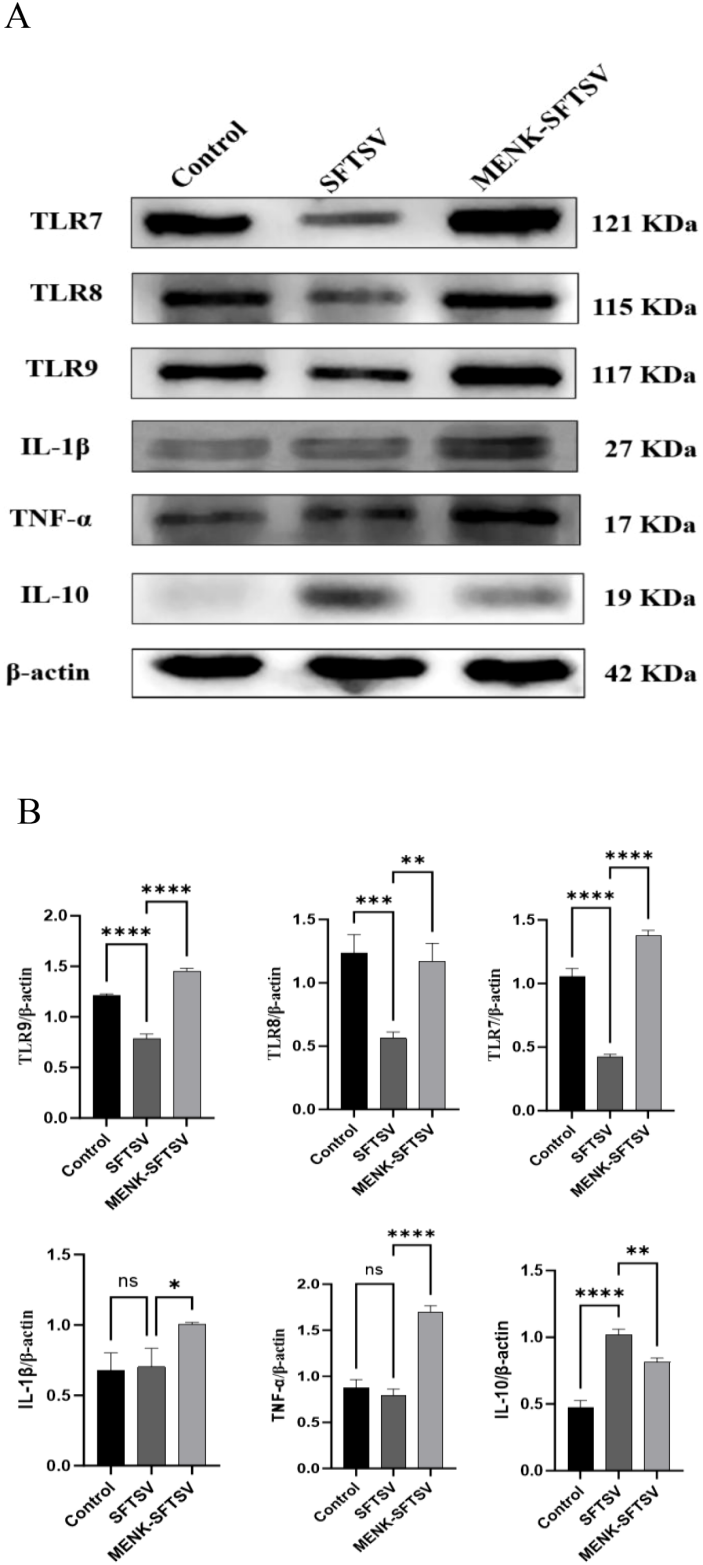
MENK upregulated TLR in RAW264.7 cells and modulated cytokine production. **(A)** Western blot analysis of TLR7, TLR8, TLR9, IL-1β, TNF-α, and IL-10 protein expression levels in the Control, SFTSV, and MENK–SFTSV groups. **(B)** Histograms of the grayscale analysis of the corresponding proteins. Control group vs. SFTSV group and SFTSV group vs. MENK-SFTSV group, *P < 0.05, **P < 0.01, ***P < 0.001, ****P < 0.0001.

The expression levels of TLR7, TLR8, and TLR9 were quantified in the three macrophage groups. Compared with the control treatment, SFTSV infection significantly downregulated the mRNA levels of TLR7, TLR8, and TLR9 (P < 0.001). By contrast, the MENK–SFTSV group exhibited markedly higher expression levels of TLR7, TLR8, and TLR9 than the SFTSV group (P < 0.01) (**Figures 11A, B**). We further examined the effect of MENK on the secretion of the proinflammatory cytokines IL-1β and TNF-α and the anti-inflammatory cytokine IL-10. Relative to the control group, the SFTSV group showed no significant changes in IL-1β and TNF-α expression levels but had markedly elevated IL-10 levels (P < 0.0001), suggesting that SFTSV infection may suppress inflammatory responses. However, compared with the SFTSV group, the MENK–SFTSV group displayed increased expression levels of the proinflammatory cytokines IL-1β and TNF-α (P < 0.05) and decreased expression of IL-10 (P < 0.01) (**Figures 11A, B**). Western blot analyses confirmed that MENK effectively upregulated the pattern recognition receptors TLR7, TLR8, and TLR9 and significantly modulated the production of IL-1β, TNF-α, and IL-10. These findings underscored the critical role of MENK in antiviral immunity and provided experimental evidence for its potential to inhibit SFTSV replication and regulate host antiviral immune responses.

### 3.12 MENK inhibited SFTSV proliferation by upregulating TLR7 in RAW264.7 cells

**Figure 12:**
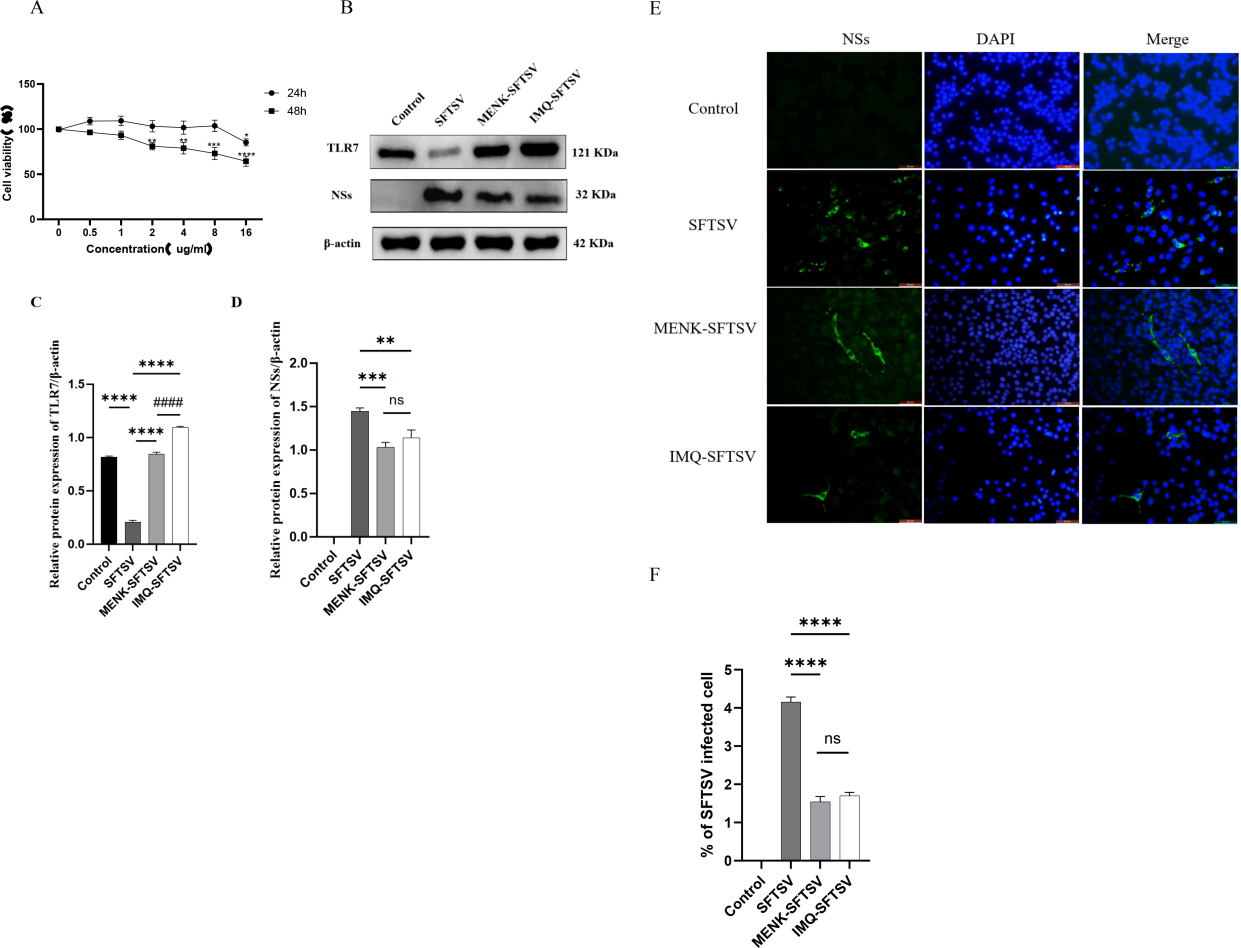
Overexpression of TLR7 can inhibit the replication of SFTSV in RAW264.7 cells. **(A)** Cytotoxicity assay of the effects of IMQ on RAW264.7 cells. Comparisons were made between cells treated with different concentrations of IMQ for 24 and 48 h and the untreated group (0 μg/mL IMQ). 24 h vs. 48 h group, **P < 0.01, ***P < 0.001, ****P < 0.0001. **(B)** Detection of TLR7 and NS expression levels in the Control, SFTSV, MENK–SFTSV, and IMQ–SFTSV groups through Western blot analysis. **(C, D)** Histograms of the grayscale analysis of TLR7 and NSs. **(E)** Expression of NSs in the Control, SFTSV, MENK–SFTSV, and IMQ–SFTSV groups detected through immunofluorescence assay (400×). Green fluorescence indicates SFTSV NSs. **(F)** Bar chart of the percentage of SFTSV-infected cells in four groups detected through immunofluorescence assay. Comparisons between the Control group and SFTSV group, SFTSV group and MENK-SFTSV group, as well as SFTSV group and IMQ-SFTSV group, **P < 0.01, ***P < 0.001, ****P < 0.0001. MENK-SFTSV vs. IMQ-SFTSV group, ^####^P < 0.0001.

We optimized the experimental conditions for the TLR7 agonist IMQ through CCK-8 assays. After 24 h, cell viability remained unchanged under treatment with 0.5–8 μg/ml IMQ but significantly reduced under treatment with 16 μg/ml IMQ (P < 0.05). Prolonging the incubation to 48 h caused a concentration-dependent decline, with cell viability in the group under 2 μg/ml IMQ treatment significantly decreasing compared with that in the untreated control group (P < 0.01) (**Figure 12A**). On the basis of these data, treatment with 8 μg/ml IMQ for 24 h was chosen as the optimal condition that effectively activated RAW264.7 cells without compromising cell viability. In the MENK–SFTSV group, RAW264.7 cells were pretreated with 10 mg/ml MENK for 48 hours (21), while the IMQ–SFTSV group received 8 μg/ml IMQ for 24 hours. Following pretreatment, both groups were infected with SFTSV at MOI 200. After 48 h of culture, Western blot and immunofluorescence analyses were performed.

Western blot analysis was performed to examine the expression levels of TLR7 and NSs in four groups of RAW264.7 cells (**Figure 12B**). Compared with the SFTSV group, the IMQ–SFTSV group exhibited a significant increase in TLR7 protein expression (P < 0.0001) (**Figure 12C**) and a marked reduction in NS levels (P < 0.001) (**Figure 12D**), indicating that TLR7 activation suppressed SFTSV proliferation in RAW264.7 cells. Notably, no statistically significant difference was observed in TLR7 and NS expression levels between the MENK–SFTSV and IMQ–SFTSV groups, suggesting that MENK exerted a TLR7 agonistic effect similar to that of IMQ, upregulating TLR7 expression in macrophages and thereby inhibiting SFTSV replication.

We performed an immunofluorescence assay to detect the expression and localization of SFTSV NSs for the further visual observation of SFTSV infection status in cells. Immunofluorescence showed intense green NS staining across most cells in the SFTSV group, confirming robust viral replication, whereas the MENK–SFTSV and IMQ–SFTSV groups exhibited markedly reduced, sporadic fluorescence (**Figure 12E**). Quantitative analysis revealed that the percentage of infected cells was significantly lower in the MENK–SFTSV and IMQ–SFTSV groups than in the SFTSV group (P < 0.0001) but did not differ between the MENK–SFTSV and IMQ–SFTSV groups (**Figure 12F**). Our imaging data provided direct evidence that MENK, like the TLR7 agonist IMQ, induced TLR7 overexpression and effectively suppressed SFTSV proliferation in RAW264.7 cells, offering a visual basis for further mechanistic studies on anti-SFTSV activity.

### 3.13 MENK demonstrated superior efficacy to IMQ in enhancing macrophage defense against SFTSV infection

**Figure 13:**
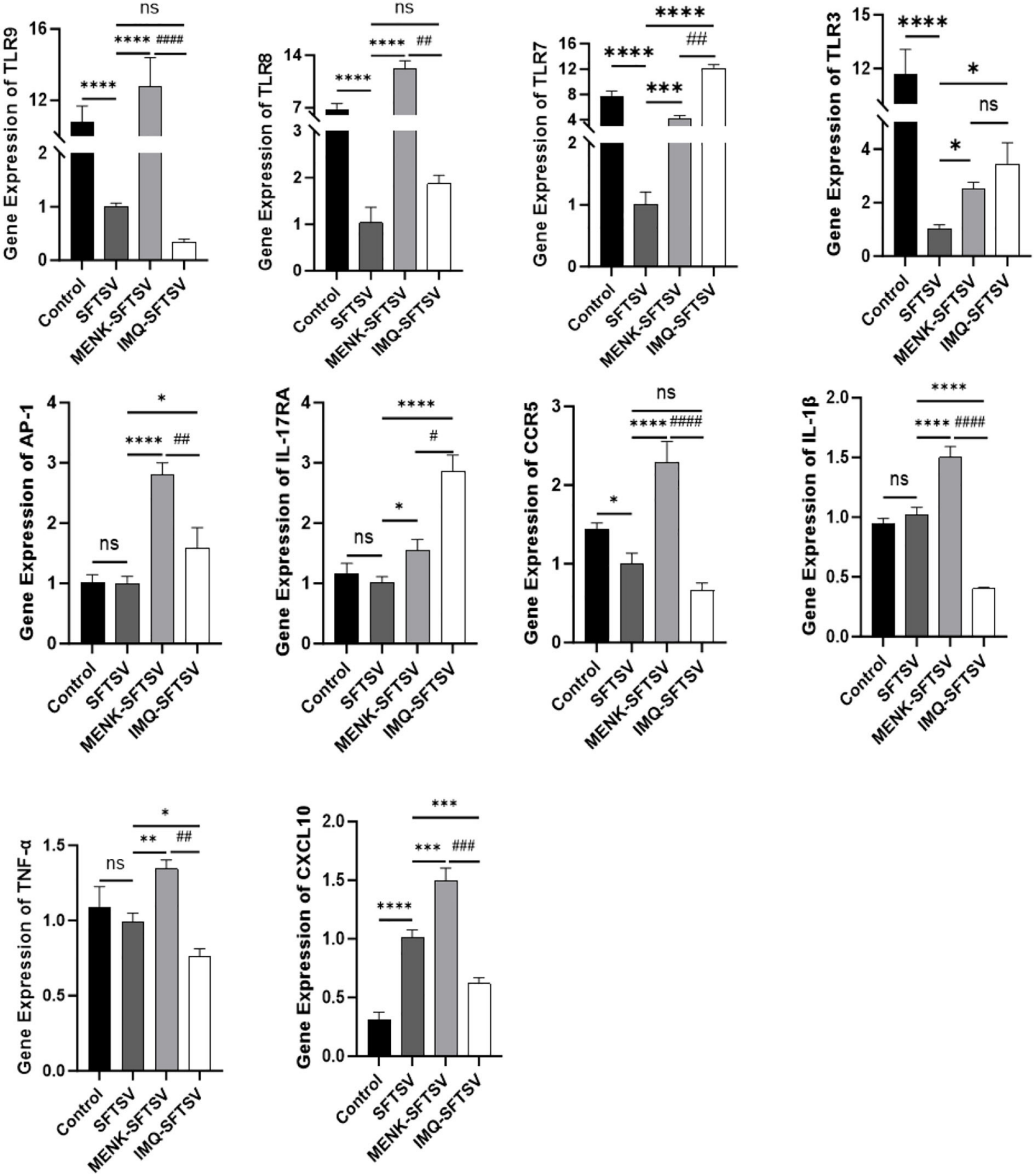
Effects of MENK and IMQ treatment on the antiviral state of RAW264.7 cells. Detection of the expression levels of TLR9, TLR8, TLR7, TLR3, AP-1, IL-17RA, CCR5, IL-1β, TNF-α, and CXCL10 in the Control, SFTSV, MENK–SFTSV, and IMQ–SFTSV groups by qPCR. Comparisons between the Control group and SFTSV group, SFTSV group and MENK-SFTSV group, as well as SFTSV group and IMQ-SFTSV group, *P < 0.05, **P < 0.01, ***P < 0.001, ****P < 0.0001. MENK-SFTSV vs. IMQ-SFTSV group, ^#^P < 0.05. ^##^P < 0.01, ^###^P < 0.001, ^####^P < 0.0001.

We performed qPCR validation on 10 significant DEGs between the MENK–SFTSV and SFTSV groups identified through RNA-seq (**Figure 13**) to compare the effects of MENK and IMQ in regulating macrophage responses against SFTSV infection. Compared with SFTSV infection, IMQ treatment significantly upregulated the expression levels of TLR7, TLR3, AP-1, and IL-17RA (P < 0.05) while downregulating those of IL-1β, TNF-α, and CXCL10 (P < 0.05). By contrast, MENK not only upregulated TLR7, TLR3, AP-1, and IL-17RA but also significantly enhanced the expression levels of TLR9, TLR8, TNF-α, IL-1β, CCR5, and CXCL10 (P < 0.01). Direct comparison revealed that the MENK–SFTSV group exhibited significantly higher expression levels of TLR9, TLR8, AP-1, CCR5, IL-1β, TNF-α, and CXCL10 than the IMQ–SFTSV group (P < 0.01). The broad-spectrum upregulation of these genes by MENK suggested that MENK exerted a more robust immunomodulatory effect than IMQ in enhancing macrophage-mediated antiviral responses against SFTSV. These findings indicated that MENK synergistically strengthened the antiviral state of macrophages through multiple pathways and regulatory mechanisms, demonstrating superior efficacy in combating SFTSV infection.

## 4 Discussion and conclusions

The prognosis of SFTSV infection, that is, whether it leads to recovery or fatal outcomes, has been shown to be correlated with the severity of cytokine storm, which is characterized by the excessive production of IL-6 and IL-10 alongside decreased TGF-β levels (28). SFTSV NSs mediate the degradation of key molecules in host immune response pathways, including RIG-I, TLR3, TNF receptor–associated factors (TRAFs), STATs, TBK1, and MAVS, thereby evading innate immune attacks (29). Macrophages, as crucial components of the innate immune defense system, play a dual role during SFTSV infection. First, viral Gn and Gc glycoproteins facilitate macrophage infection through receptor attachment and membrane fusion. Second, macrophages can recognize vRNA via TLRs (30), subsequently activating MyD88-dependent signaling pathways to promote cytokine release (31) and trigger antiviral responses. Developing novel strategies to ameliorate severe immune dysregulation and innate immune suppression caused by SFTSV infection is critical for vaccine and drug development. Previous studies by our group demonstrated that MENK enhanced macrophage-mediated anti-influenza virus responses by upregulating the TLR4–NF-κB p65 pathway and promoting the expression of opsonizing receptors and chemokines (21, 22). By building on these findings, we employed RNA-seq technology to elucidate the mechanisms by which MENK regulates macrophage defenses against SFTSV infection, providing new perspectives and clues for clinical SFTSV management. Experimental strategy and key findings regarding MENK’s regulation of macrophage-mediated anti-SFTSV responses is provided in **Supplementary Figure S7**.

RNA-seq data revealed that the expression levels of TLRs, particularly TLR7, TLR8, and TLR9, in the SFTSV-infected group significantly reduced relative to those in the Control group, suggesting that SFTSV may achieve immune evasion by suppressing pattern recognition receptors in macrophages. During coevolution with their hosts, many viruses have evolved sophisticated escape strategies, such as degrading TLR signaling components, disrupting complex assembly, interfering with transcription factor activation, mimicking cellular proteins, or deubiquitinating key adaptors, thereby blunting TLR-triggered defenses (32). NS3/4A serine protease from the hepatitis C virus cleaves TRIF (33), and vaccinia virus A46R binds MyD88, TRIF, and TRAM via its TIR domain to prevent signalosome formation. TLRs and Interleukins are involved in maintaining homeostasis and play significant roles in infectious diseases. Any mutations or single nucleotide polymorphisms (SNPs) occurring in interleukin genes may lead to various diseases and dysregulations (34). TLR SNPs are associated with poor antiviral responses against SARS-CoV-2 (35). TLR agonists can selectively enhance anti-SARS-CoV-2 host immunity to reduce the risk of infection (36). The activation of macrophage TLRs normally induces cytokine expression through AP-1 and NF-κB, enabling cytotoxic lymphocytes to control infection (37). Importantly, our data showed that MENK restored TLR7, TLR8, and TLR9 expression levels in SFTSV-infected macrophages (ko04620), reversing viral repression. This TLR upregulation enhances viral recognition, antiviral effector functions, and antigen presentation, thereby strengthening the macrophage-mediated response against SFTSV.

Moreover, comparative RNA-seq between MENK–SFTSV and SFTSV macrophages revealed that DEGs were enriched in IL-17 signaling (ko04657), TNF signaling (ko04668), TLR signaling (ko04620), and virus cytokine/chemokine interactions (ko04061). Notably, elevated AP-1 levels were consistently observed across these enriched pathways. AP-1, a heterodimeric transcription factor composed of c-Fos and c-Jun, plays pivotal roles in regulating cellular differentiation, migration, proliferation, and apoptosis (38). Previous studies have demonstrated that porcine reproductive and PRRSV upregulate IL-1β in microglia by modulating AP-1, MyD88, and ERK expression (39). The SARS-CoV-2 delta spike protein activates NF-κB and AP-1 signaling in human THP1 monocytes, inducing proinflammatory cytokine release (TNF-α, IL-1β, and IL-6) from macrophages and dendritic cells (40). Therefore, in our study, the upregulation of AP-1 likely enhanced immune defense by binding cognate regulatory elements to drive the secretion of immune mediators, recruit effector cells to infection sites, and amplify antiviral defense. These findings collectively suggested that MENK-mediated AP-1 activation strengthened antiviral responses through coordinated cytokine production and immune cell recruitment, offering a potential mechanism for its protective effects against SFTSV infection.

In terms of signaling pathways, our findings revealed a functional interplay between the TLR (ko04620) and TNF (ko04668) signaling pathways. Following ligand recognition, TLR7 recruits the adaptor protein MyD88, initiating a downstream signaling cascade that activates transcription factors such as NF-κB and AP-1. This, in turn, promotes the expression of pro-inflammatory cytokines, including TNF-α, IL-1β, and MIP-1, thereby orchestrating innate immune and inflammatory responses (41, 42). The upregulated TNF-α subsequently activated the TNF signaling pathway (ko04668), stimulating downstream effectors, including AP-1, TRAF1, and NF-κB. This cascade promoted the enhanced secretion of the proinflammatory cytokines IL-1β and CXCL10 from macrophages and neutrophils, thereby amplifying the inflammatory response. Liniger M et al. demonstrated that TNF limited the replication of the classical swine fever virus in PEDSV.15 cells by inducing IRF1-dependent type I IFN responses (43). Ito M et al. reported that 24 h TNF-α pretreatment inhibited varicella zoster virus replication in human embryonic lung fibroblasts (44). TNF-α serves as a potent NF-κB inducer, regulating the nuclear expression of multiple proinflammatory genes (45). TRAF1, as a key adaptor molecule in the TNFR, TLR, cytokine, and antigen receptor signaling pathways, plays pivotal roles in immune regulation (46). Zhi L et al. (47) demonstrated that in orange-spotted grouper, TRAF interacts with tripartite motif proteins, modulating immune responses against iridovirus and nodavirus infections. The elevated IL-1β synergizes with TNF-α to enhance immune cell activation, stimulate dendritic cell maturation, and promote adaptive immune responses (48). Thompson AJ et al. (49) further showed that IL-1β combined with treatment with the TLR2 ligand Pam-2-Cys suppresses HBV replication in Huh-7 hepatoma cells. CXCL10 (IP-10) further augments defense by recruiting neutrophils and macrophages to infection foci, activating NK cells and modulating cytokine release (50). Critically, IP-10 restricts the zika virus in human prostate cells (51) and has additional protective roles in SARS coronavirus infection (52) and EBV-immortalized normal cells (53). Collectively, our data indicated that the MENK pretreatment of macrophages drove the robust secretion of cytokines and chemokines during SFTSV challenge, thereby intensifying inflammatory responses and enhancing effective antiviral immunity against SFTSV.

In the IL-17 signaling pathway (ko04657), we observed significant alterations in IL-17RA expression. IL-17RA upregulation, which is activated by cytokines, including IL-17 and IL-1β, promotes neutrophil recruitment and enhances immune responses against extracellular pathogens (54). IL-17RA plays a pivotal role in mediating the pathogenic mechanisms of various microbes, including the influenza virus and *Klebsiella pneumoniae*, and mycoplasma infections (55). Mechanistically, as demonstrated by Li JK et al. (56), IL-17A enhances the expression of the inflammatory mediators cyclooxygenase-2 (COX2) and prostaglandin E2 in nucleus pulposus cells via the p38–c-Fos and JNK–c-Jun pathways, thereby modulating inflammatory responses. In this pathway, MENK-activated IL-17RA triggers a downstream cascade. IL-17RA signaling orchestrates a multilayered antiviral strategy by upregulating antiviral cytokines, such as IL-1β, TNF-α, COX2, and GM-CSF, to drive immunity while simultaneously inducing the chemokine CXCL10 to mediate cellular recruitment, thereby efficiently counteracting viral invasion.

Furthermore, by comparing the qPCR results of MENK with those of the TLR7 agonist, we found that the TLR7 agonist IMQ upregulated the expression levels of TLR7, TLR3, AP-1, and IL-17RA, whereas MENK not only upregulated these four genes but also significantly enhanced the expression levels of TLR9, TLR8, IL-1β, TNF-α, CCR5, and CXCL10. TLRs categorize into two groups, viral and non-viral TLRs. TLR1, 5, and 6 are associated with non-viral ligands, while TLR2, 3, 4, 7, 8, 9, and 10 are considered to be viral ligand-related. MENK concurrently upregulated the levels of TLR3, TLR7, TLR8, and TLR9 in RAW264.7 cells. These results indicated that MENK possesses unique advantages and exhibits efficacy comparable to TLR agonists in modulating the antiviral state of macrophages. Rather than relying on a single signaling axis, MENK engaged multiple targets and pathways, thereby exceeding the scope of single-receptor agonists and conferring macrophages with a broad and robust antiviral state that affords the host exceptional protection against viral infections. For subsequent experiments, we will include TLR antagonists to further validate the role of TLRs in enhancing host immunity and reducing viral infection risk, as well as to investigate if the superior performance of MENK as an immunomodulatory peptide in upregulating TLR-associated pathways, notably the MyD88-dependent and MAPK signaling pathways.

The key strength of this study lay in its systematic elucidation of the unique mechanism by which MENK counteracts SFTSV infection through synergistic multi-target and multi-pathway actions. Unlike single-target TLR agonists, MENK not only reversed viral suppression of key pattern recognition receptors such as TLR7/8/9 but also modulated the cytokine network to enhance antiviral effects. It should be noted that the ability of MENK to suppress SFTSV infection, as revealed in this study, remains at a preliminary stage of mechanistic exploration. Key functional assessments of MENK treated macrophages require further analysis, such as the verification of AP-1 nuclear translocation and a comprehensive evaluation of macrophage activation status. Future studies will employ a multifaceted approach to deepen our understanding of macrophage activation. This will include a functional assessment of macrophage activity through measurements of respiratory burst, co-stimulatory markers, and MHC I expression, coupled with a systematic analysis of the immune response at the gene and protein levels. Additionally, we will utilize highly sensitive cytokine profiling techniques (e.g., NULISA, Luminex) followed by TaqMan probe-based validation of critical targets to achieve high specificity data with reduced background interference.

Taken together, our results demonstrated that MENK enhanced antigen recognition by upregulating TLRs; promoted the expression of transcription factors, such as AP-1 and NF-κB; and activated downstream signal transduction, enabling macrophages to maintain a highly efficient transcriptionally activated state to meet immune defense demands. It stimulated the secretion of proinflammatory cytokines and chemokines while suppressing the release of the anti-inflammatory cytokine IL-10, forming a positive feedback loop that enhanced the macrophage-mediated killing of SFTSV. Our observations further emphasized that MENK exhibited immunostimulatory effects via the multitargeted upregulation of TLRs and inflammatory pathways, effectively counteracting SFTSV-induced TLR suppression to establish a robust antiviral state in macrophages. By restoring macrophage antiviral function and overcoming viral immune evasion mechanisms, MENK emerges as a promising host-directed therapeutic candidate or vaccine prototype against SFTSV infection, warranting further preclinical and clinical investigation.

## Data Availability

The original contributions presented in the study are publicly available. This data can be found here: [https://www.ncbi.nlm.nih.gov/bioproject/PRJNA1359617/].

## Supplementary material

Supplementary Figure 1 Viability of SFTSV-infected RAW264.7 cells as determined by CCK-8 assay.

Supplementary Figure 2 KEGG pathway enrichment map of neutrophil extracellular trap formation (ko04613).

Supplementary Figure 3 KEGG pathway enrichment map of the IL-17 signaling pathway (ko04657).

Supplementary Figure 4 KEGG pathway enrichment map of the TLR signaling pathway (ko04620).

Supplementary Figure 5 KEGG pathway enrichment map of the TNF signaling pathway (ko04668).

Supplementary Figure 6 KEGG pathway enrichment map of viral protein interaction with cytokines and cytokines receptor (ko04061).

Supplementary Figure 7 Research workflow summarizing the experimental strategy and key findings.
