# Supplementary figures and images for: Methionine enkephalin upregulates toll-like receptors in macrophages to suppress severe fever with thrombocytopenia syndrome virus infection
Source: Front Immunol. 2025 Nov 18;16:1700988. doi: 10.3389/fimmu.2025.1700988 (PMC12746651; doi:10.3389/fimmu.2025.1700988)

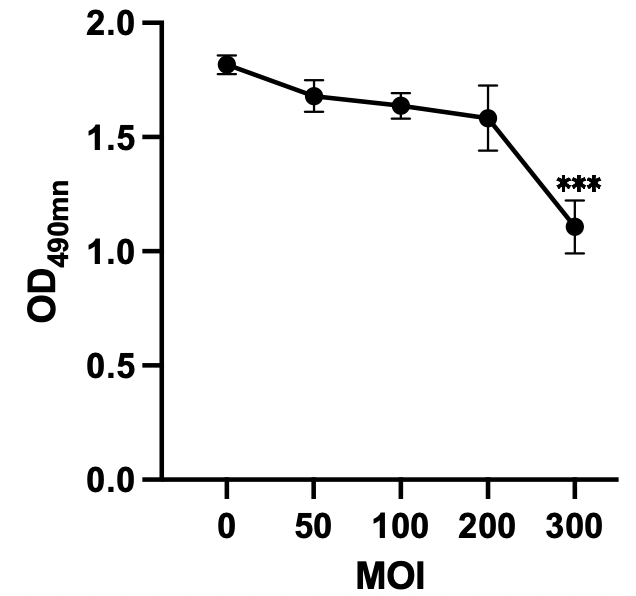

Supplement: Supplementary Figure 1 — Viability of SFTSV-infected RAW264.7 cells as determined by CCK-8 assay. [file Image1.png]

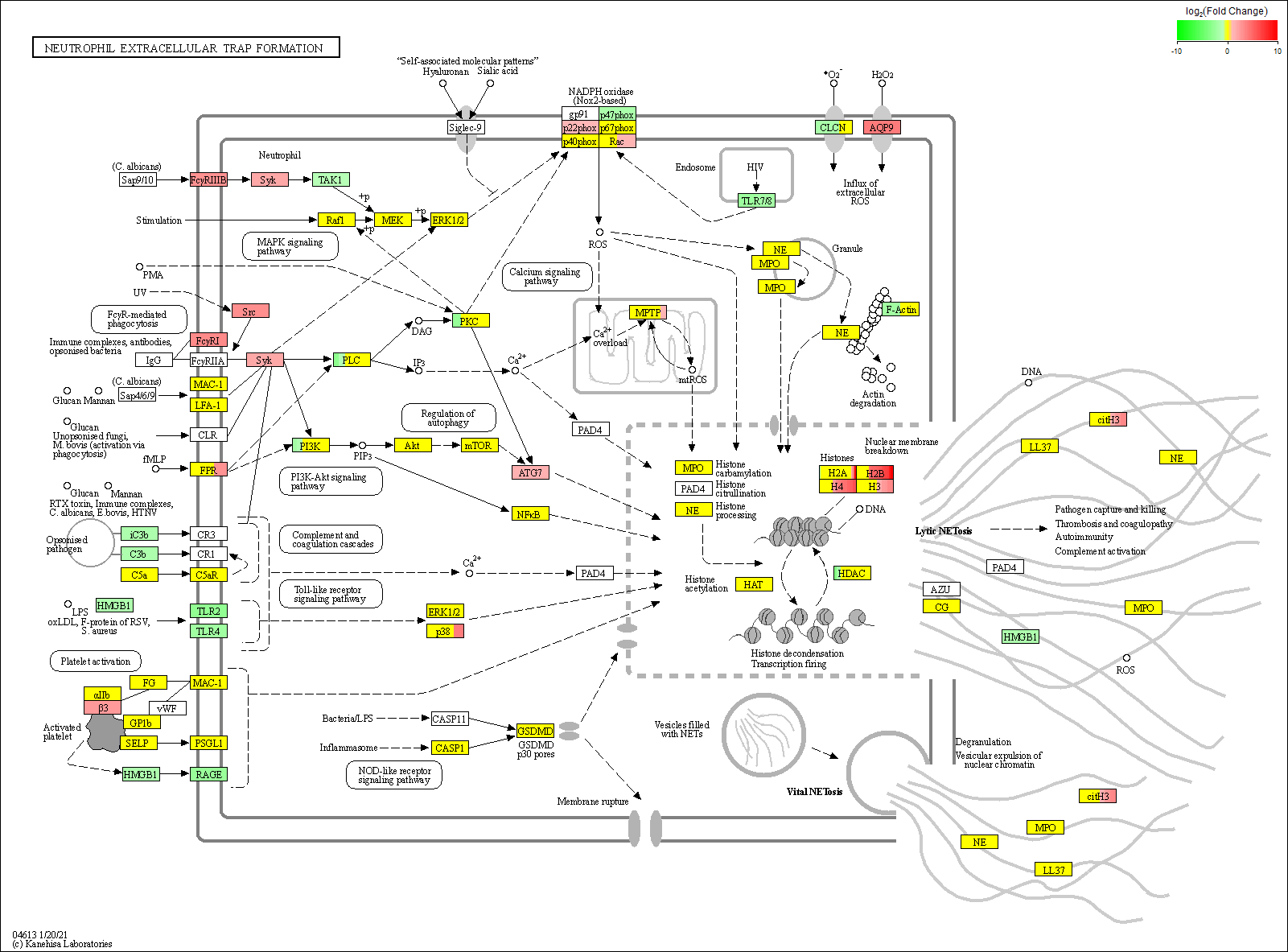

Supplement: Supplementary Figure 2 — KEGG pathway enrichment map of neutrophil extracellular trap formation (ko04613). [file Image2.png]

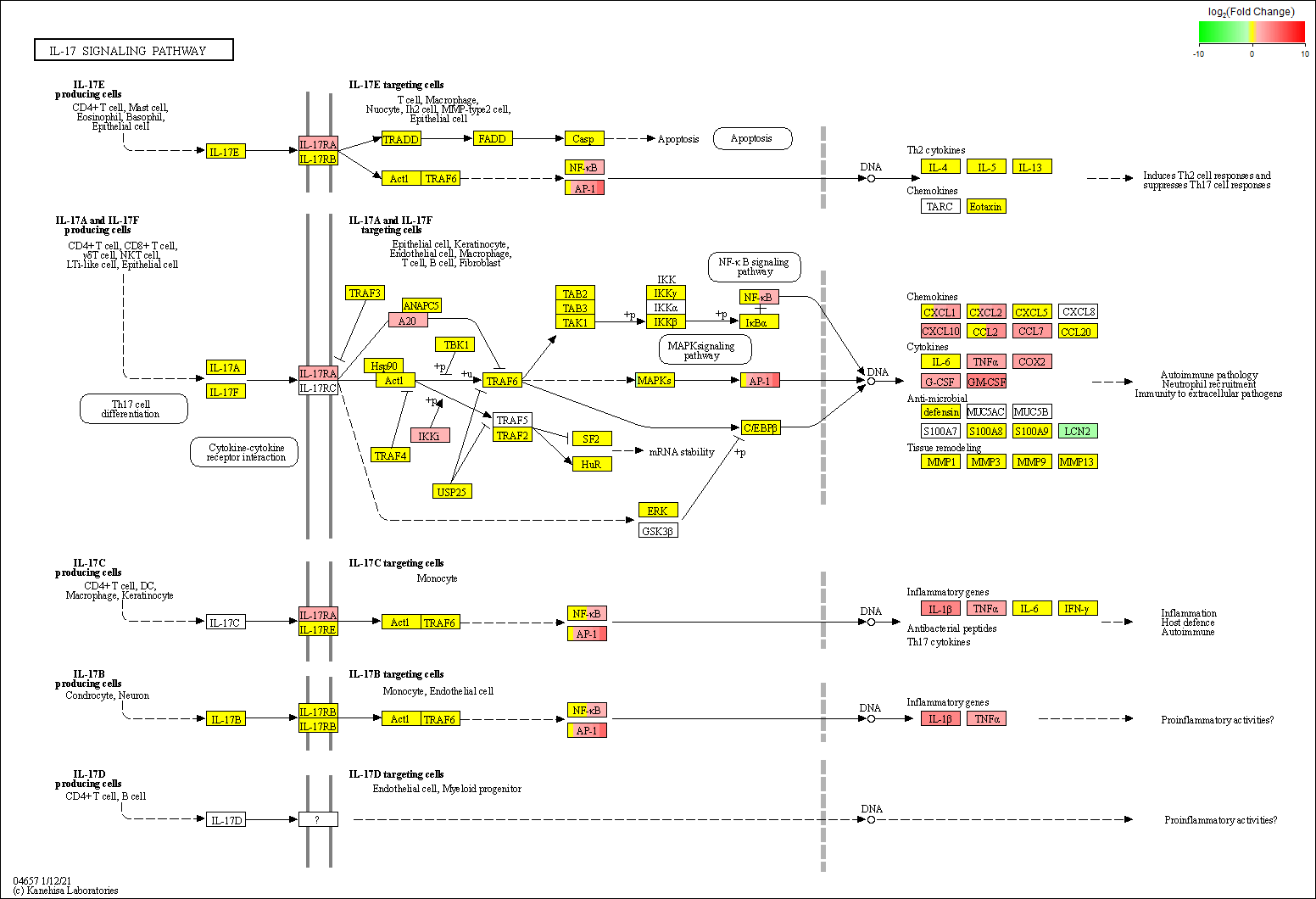

Supplement: Supplementary Figure 3 — KEGG pathway enrichment map of the IL-17 signaling pathway (ko04657). [file Image3.png]

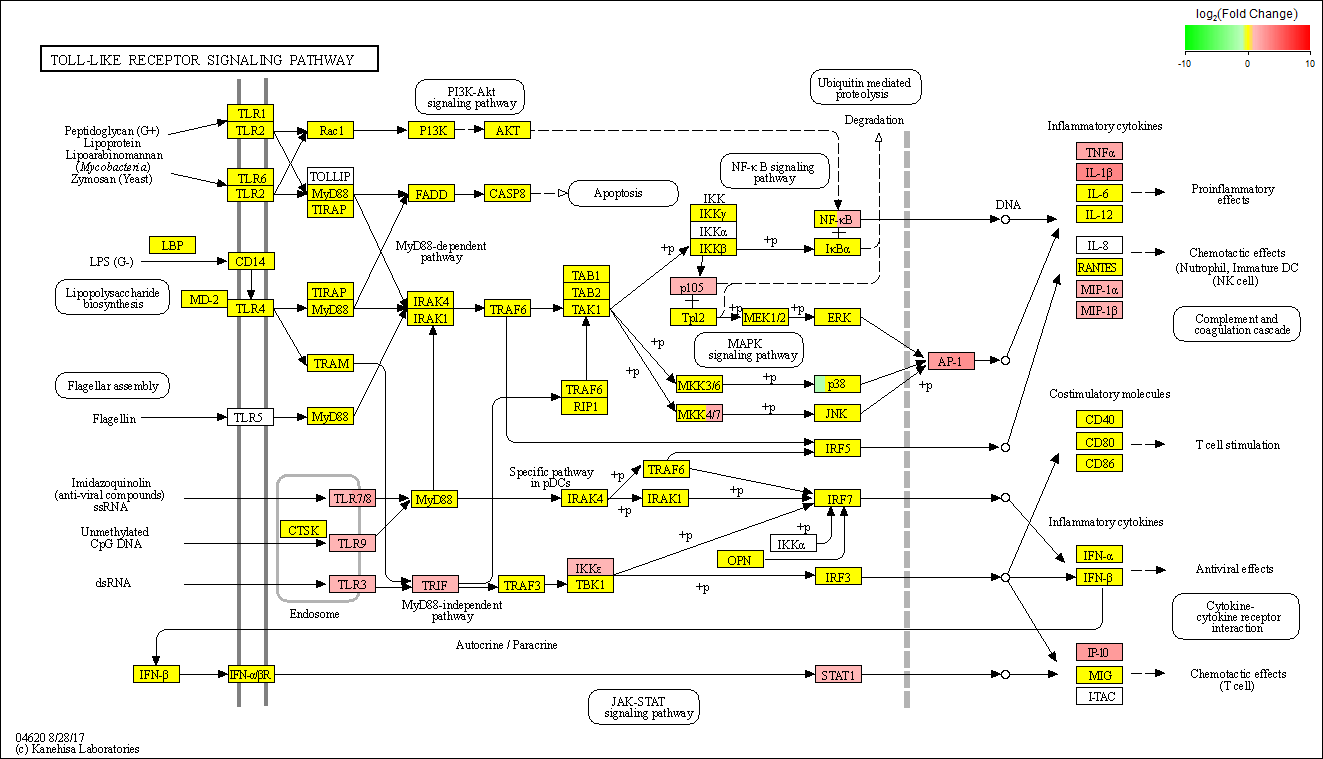

Supplement: Supplementary Figure 4 — KEGG pathway enrichment map of the TLR signaling pathway (ko04620). [file Image4.png]

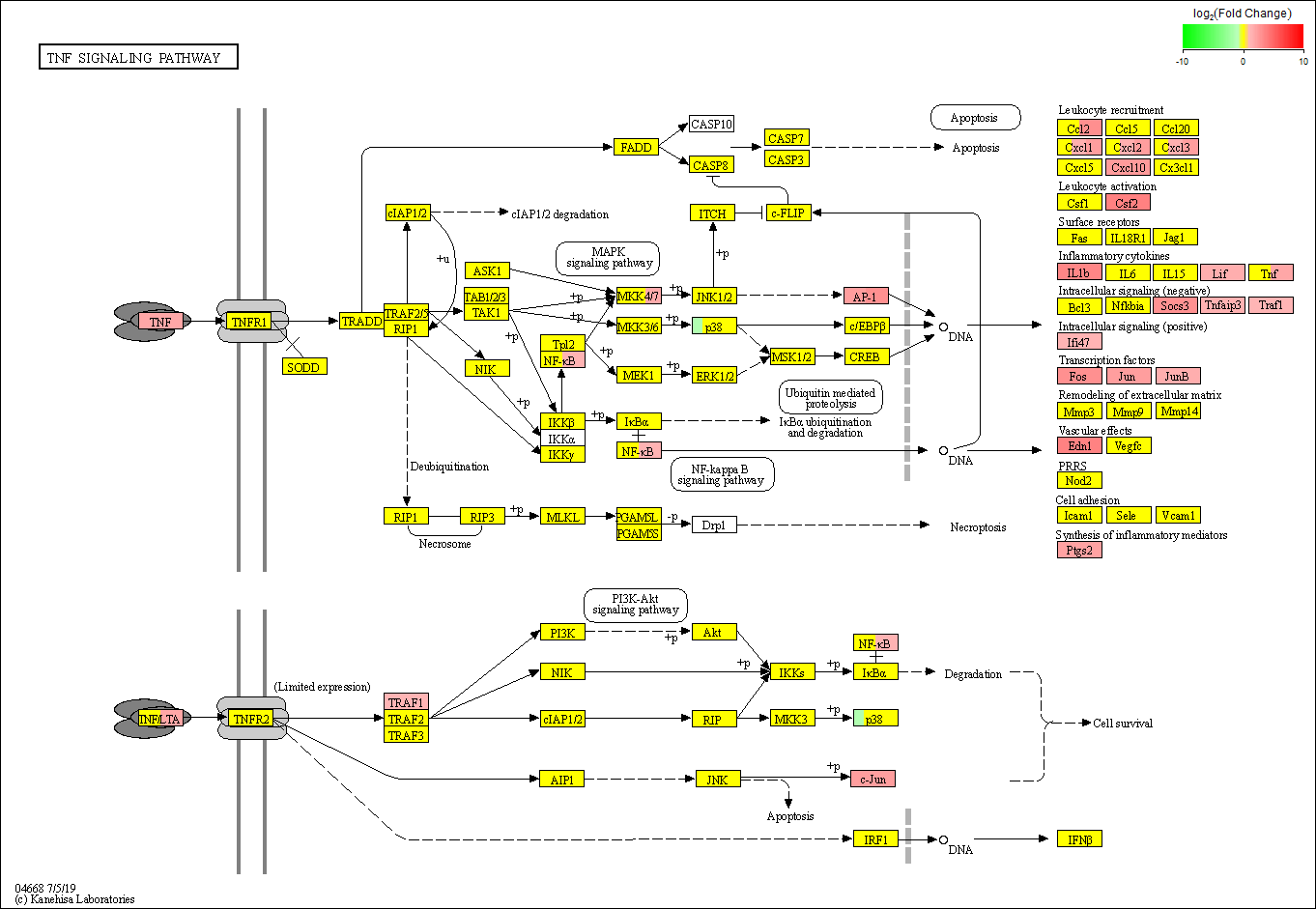

Supplement: Supplementary Figure 5 — KEGG pathway enrichment map of the TNF signaling pathway (ko04668). [file Image5.png]

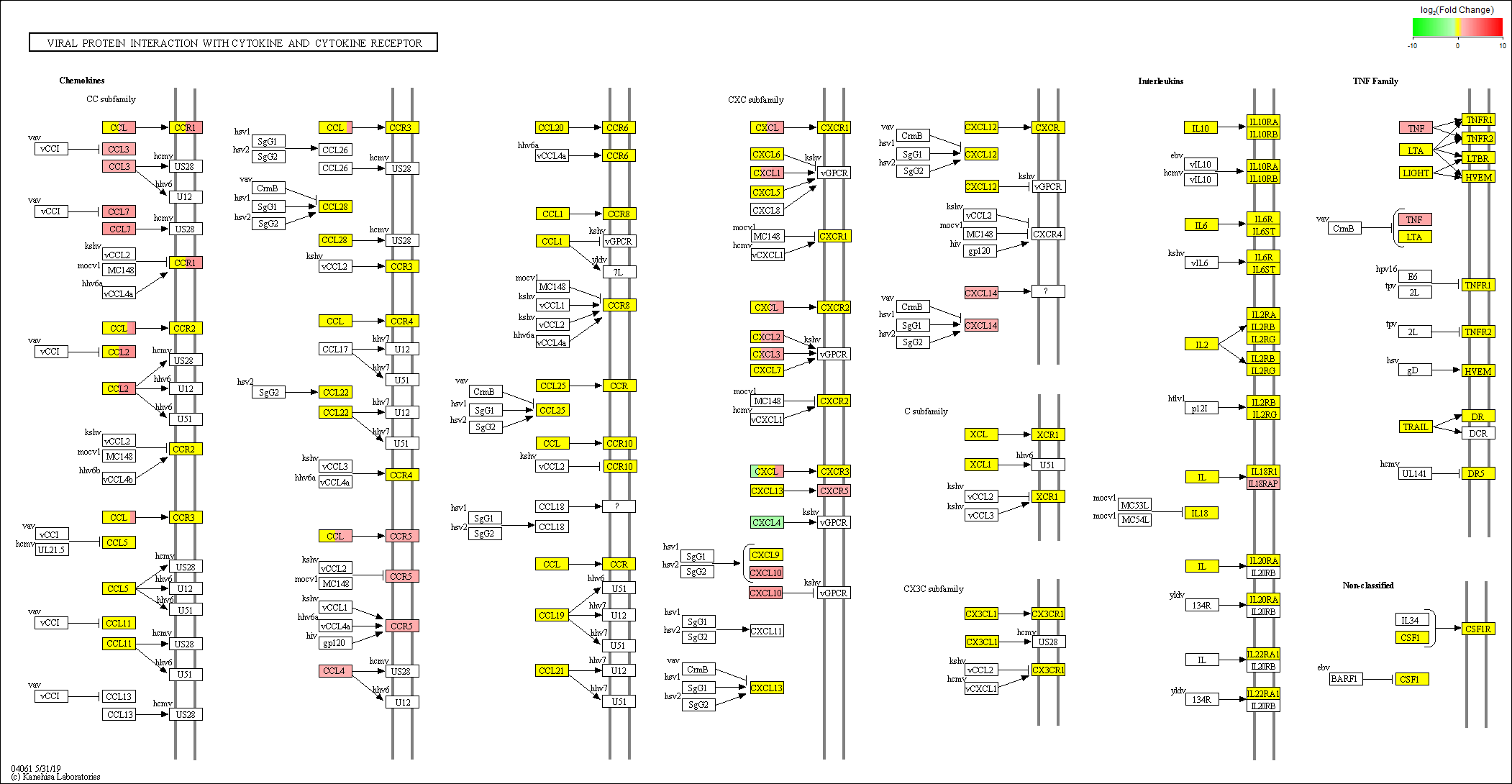

Supplement: Supplementary Figure 6 — KEGG pathway enrichment map of viral protein interaction with cytokines and cytokines receptor (ko04061). [file Image6.png]

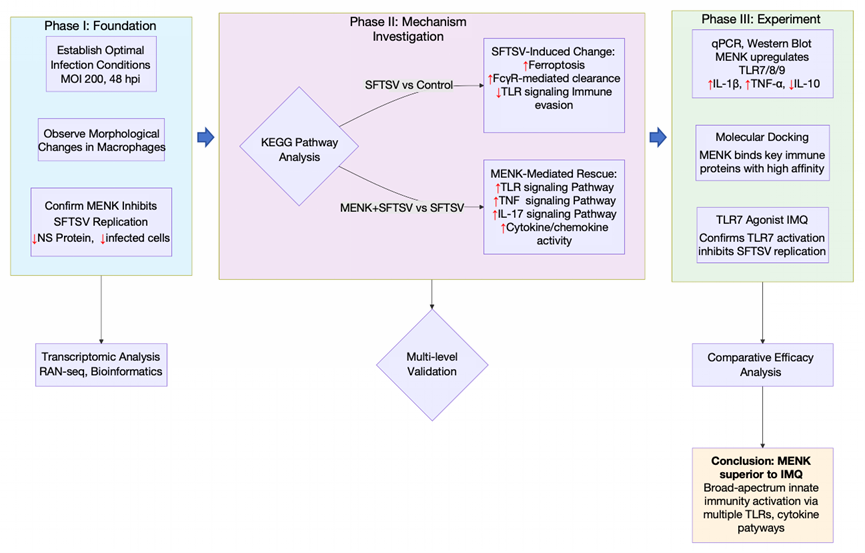

Supplement: Supplementary Figure 7 — Research workflow summarizing the experimental strategy and key findings. [file Image7.png]
